# Competitive Adsorption Mechanisms of Cu(II) and Cd(II) on Mineral–Humic Acid–*Pseudomonas putida* Composites: Implications for Heavy Metal Retention in Agricultural Soils

**DOI:** 10.3390/toxics14090743

**Published:** 2026-08-23

**Authors:** Guang Hao, Min Xiao, Shifeng Li, Dongmei Zheng, Ying Ji, Huiying Li, Xin Yang, Ruiying Bu, Wanlin Xian, Yinggang Wang

**Affiliations:** 1 Key Laboratory of Ecological Restoration of Regional Contaminated Environment, Ministry of Education, College of Environment, Shenyang University, Shenyang 110044, China; hedgehog2000@163.com (G.H.); zhengdm126@163.com (D.Z.); syu0240806@163.com (Y.J.); tjlhy299@126.com (H.L.); yx15504026203@163.com (X.Y.); 15213905572@163.com (R.B.); 13065301081@163.com (W.X.); 2 College of Chemical Engineering, Shenyang University of Chemical Technology, Shenyang 110142, China; li.shi.feng@163.com

**Keywords:** competitive adsorption, heavy metal, clay mineral, humic acid, *Pseudomonas putida*, interfacial interactions

## Abstract

The fate of heavy metals in agricultural soils is governed by organo-mineral–microbial interactions, which predictive models often fail to capture. The competitive sorption mechanisms of Cd(II) and Cu(II) on montmorillonite/kaolinite composites (Mont/Kao) functionalized with humic acid (HA) and *Pseudomonas putida* (*P. p*), a model system representative of contaminated agricultural soils, were investigated. Batch experiments, XRD, FTIR, and thermodynamic analysis reveal that metal retention is a non-additive function of competing interfacial processes. Bacterial biomass dominated sorption, accounting for >50% of total metal uptake, with capacity ranked as: *P. p* > Mont/Kao-*P. p* > Mont/Kao-HA-*P. p* > Mont/Kao-HA > Mont/Kao. Humic acid exerts a dual, concentration-dependent role: Low levels enhanced adsorption via mineral dispersion, while high levels induced surface masking, suppressing bacterial binding sites. Competition was highly asymmetric: Cd(II) reduced Cu(II) maximum adsorption capacity by 75.5% in the Mont/Kao-HA system by preferentially occupying montmorillonite interlayer sites, whereas Cu(II) inhibited Cd(II) below pH 6. Single-metal sorption was characterized by positive Δ*S*° (32.96–58.89 J·mol^−1^·K^−1^), indicative of inner-sphere complexation, while negative Δ*S*° under competitive conditions signals a transition to outer-sphere complexation. This work provides mechanistic insights into site masking, competitive displacement, and ternary cation bridging controlling metal immobilization in organo-mineral assemblages.

## 1. Introduction

The rapid intensification of agricultural production in China, characterized by the long-term application of chemical fertilizers, pesticides, and organic amendments, together with elevated geogenic background levels, has resulted in the accumulation of heavy metals (HMs) in agricultural soils. Among these contaminants, Cd is recognized as one of the primary pollutants affecting soil environmental quality, followed by As, Cu, Ni, and Pb [1,2]. Although nationwide monitoring indicates a declining trend in overall soil HM concentrations, contamination remains a persistent concern in certain agricultural regions [3]. This issue is particularly relevant in greenhouse agricultural systems, where intensive cultivation and repeated external inputs may accelerate HM accumulation and increase potential risks to food safety, ecosystem stability, and human health.

In natural soil environments, HM mobility and retention are strongly regulated by heterogeneous colloidal components, including clay minerals, natural organic matter (NOM), microorganisms, and their extracellular products [4,5,6]. Individual soil components have been extensively investigated. Fundamental adsorption mechanisms, including ion exchange, surface complexation, and ligand-mediated coordination, have been elucidated in previous studies [7,8,9], and most existing investigations have been restricted to simplified binary systems, such as clay–metal, humic acid–metal, or bacteria–metal interactions. However, these components rarely exist independently in soils. Instead, they interact through complex physicochemical processes to form heterogeneous organo-mineral assemblages, in which interfacial interactions may substantially modify the accessibility and reactivity of metal-binding sites [10,11]. Consequently, the mechanisms governing HM behavior in multi-component soil interfaces cannot be fully predicted based solely on the properties of individual constituents.

Although increasing evidence has demonstrated that mineral surface properties can be substantially modified by humic coatings and microbial attachment, the resulting effects have predominantly been interpreted based on the additive contributions of individual components. However, upon formation of mineral–organic–microbial interfaces, reactive site accessibility may be altered, new metal-binding environments may be generated, and competitive occupation of adsorption domains may be induced. Consequently, whether heavy metal retention in composite systems is controlled by additive adsorption capacities or governed by emergent interfacial mechanisms remains unresolved.

Recent studies have revealed that HM adsorption in organo-mineral composites frequently deviates from the additive behavior expected from individual components, demonstrating the prevalence of non-additive interfacial effects [12,13,14]. Such deviations are mainly attributed to changes in reactive site accessibility, the formation of organic and/or inorganic surface coatings that may block or modify mineral reactive sites, and cooperative interactions among minerals, organic matter, and microorganisms. Humic acid (HA) coatings can enhance Cd retention on iron minerals by providing additional complexation sites, whereas excessive organic coatings may reduce metal accessibility by masking reactive surfaces [15,16,17]. Similarly, mineral–bacterial associations can either enhance Cu adsorption by introducing additional functional groups or decrease adsorption efficiency when site masking hinders access to binding domains [18,19,20]. These findings suggest that the effects of individual components are not simply additive after composite formation, and that interfacial configuration plays a critical role in determining HM retention. However, these studies have generally examined either organic–mineral interactions or mineral–microbial associations separately. The combined effects of HA and bacteria on mineral surfaces remain poorly understood, particularly regarding how organic coatings regulate bacterial attachment, how microbial functional groups compete with mineral adsorption sites, and how these coupled processes influence metal partitioning under multi-metal conditions.

More importantly, contaminated agricultural soils commonly contain multiple heavy metals simultaneously [15,21,22]. Nevertheless, competitive adsorption between coexisting metals within mineral–humic acid–bacterial interfaces has rarely been investigated. The mechanisms controlling selective occupation, displacement, and redistribution of metals among different binding domains remain largely unknown.

Competitive interactions may alter metal selectivity, binding pathways, and immobilization efficiency. Although selectivity sequences such as Pb > Cu > Cd ≈ Zn > Ni have been reported for individual mineral systems [23,24,25,26], these relationships may change substantially within complex mineral–organic–microbial matrices. Differences in metal properties, including hydrated radius, electronegativity, and affinity toward oxygen-containing ligands, further complicate competitive adsorption behavior. Cu(II) and Cd(II) provide an ideal model system for addressing these questions because they commonly coexist in agricultural soils affected by anthropogenic inputs but exhibit contrasting interfacial behaviors. Cu(II) was reported to preferentially complex with organic functional groups through inner-sphere complexation, while Cd(II) showed a greater affinity for permanent-charge mineral sites [27]. Their asymmetric competitive adsorption further made this pair suitable for investigating metal distribution at mineral–organic–microbial interfaces. Their coexistence therefore provides an opportunity to explore how competing metals redistribute between mineral, organic, and microbial binding domains.

Moreover, organic matter and microbial extracellular polymers may modify metal partitioning by altering surface accessibility and interfacial complexation pathways, including the formation of ternary cation-bridge complexes [28,29]. Humic acid content, mineral composition, and microbial surface properties may jointly determine whether composite systems promote or inhibit HM immobilization. Previous studies have proposed that the balance between organic functional groups and mineral reactive sites influences non-additive adsorption behavior [30,31]. Nevertheless, the contribution of microorganisms, the evolution of interfacial coordination mechanisms, and the competitive partitioning of coexisting HMs within multi-component composites remain largely unresolved. Rather than simply evaluating adsorption capacities, understanding the competitive mechanisms governing metal retention requires resolving how different interfacial components interact and reorganize adsorption sites within composite systems.

To address these knowledge gaps, this study establishes a model mineral–humic acid–microbial interface consisting of montmorillonite/kaolinite, humic acid, and *Pseudomonas putida* to simulate key reactive components of agricultural soils. Unlike previous studies focusing on isolated clay-organic or clay-microbial interactions, this work systematically reconstructs a hierarchical composite system and investigates competitive Cu(II)/Cd(II) adsorption across different interfacial domains. Specifically, this study aims to: (1) reveal whether metal retention in mineral–organic–microbial composites follows additive or emergent adsorption behavior; (2) identify the dominant adsorption domains and interfacial coordination mechanisms controlling Cu(II)/Cd(II) competition; (3) elucidate how humic acid and bacterial biomass regulate metal redistribution between mineral surfaces and biological interfaces.

By integrating adsorption experiments, spectroscopic characterization, and thermodynamic analysis, this work provides mechanistic insights into non-additive heavy metal immobilization processes in complex soil interfaces and advances the prediction of metal fate in contaminated agricultural environments.

## 2. Materials and Methods

### 2.1. Materials

Agricultural soils were collected from four locations in Liaoning Province, China: (I) the Long-term Experimental Station of Shenyang Agricultural University (41°49′ N, 123°57′ E), including vegetable greenhouse, vegetable farmland, and corn farmland soils; (II) Shenbei New District, Shenyang City (42°7′ N, 123°33′ E), including corn farmland, rice farmland, and natural forest soils; (III) Fushun City (41°54′ N, 124°07′ E), including vegetable greenhouse soils, and corn farmland soils; and (IV) Panjin City (41°21′ N, 122°11′ E), including reed wetland soils, and vegetable farmland soils. The 22 vegetable greenhouses included in the study had been continuously cultivated for 3–25 years.

At each sampling site, defined as a combination of location and land-use type, three independent composite soil samples were collected as field replicates. Each composite sample consisted of five subsamples collected from the 0–20 cm surface layer of soils using the five-point sampling method. The subsamples were thoroughly homogenized and air-dried. For characterization of the naturally occurring clay mineral fraction, soil organic matter was removed via the potassium bromate (KBrO_3_) oxidation method, free iron oxides were removed using the sodium dithionite-citrate-bicarbonate (DCB) method, and calcium carbonate was removed with dilute hydrochloric acid. These pretreated samples were then ground, passed through a 300-mesh sieve, oven-dried, and stored in a desiccator until further analysis. XRD, SEM, and FTIR were used to characterize the mineral composition, surface morphology, and surface functional groups of the soil samples, respectively.

Montmorillonite (Mont) and Kaolinite (Kao), purchased from Shanghai Yuanye Bio-technology Co., Ltd. (Shanghai, China), were used as the clay minerals and ground to pass through an 80-mesh sieve. To prepare the mineral suspensions, 50 g of each clay was dispersed in 50 mL of Milli-Q water in a 1000 mL beaker. For the removal of organic matter, 15 mL of hydrogen peroxide (30% solution) was added to each suspension, with residual H_2_O_2_ subsequently eliminated by evaporation [3]. The suspension pH was adjusted to10.0 for Mont and 9.0 for Kao using 0.1 mol∙L^−1^ NaOH, followed by ultrasonication for 30 min to ensure dispersion. Following ultrasonication, each dispersion was brought to a final volume of 1000 mL with Milli-Q water, and the <2 μm colloidal fractions were isolated by sedimentation using the pipette method [32]. To recover the colloidal particles, the separated dispersions were flocculated with 0.5 mol∙L^−1^ CaCl_2_ solution. The resulting particles were washed with Milli-Q water until testing with AgNO_3_ solution confirmed the absence of chloride. Prior to subsequent utilization, the recovered minerals were dried at 60 °C, ground to pass through a100-mesh sieve, and stored.

Humic acid (HA), commercially available as a humic acid sodium salt, was purchased from Shanghai Yuanye Bio-technology Co., Ltd. (Shanghai, China). For HA extraction, the commercial peat was dissolved in 1.0 mol∙L^−1^ NaOH, and the resulting solution was filtered through a 0.45 µm filter three times. To isolate HA from the filtrate, we adjusted the pH to below 1.5 using 1.0 mol∙L^−1^ HCl, inducing HA precipitation; the precipitate was recovered by centrifugation and rinsed with Milli-Q water to remove excess salt. For preparation of the stock solution, the purified HA was dissolved in 1.0 mol∙L^−1^ NaOH and filtered through a 0.45 µm filter three times before being stored at 4 °C. Prior to use, HA working solutions were obtained by diluting the stock solution with 0.01 mol∙L^−1^ NaNO_3_ electrolyte, followed by dispersion in an ultrasonic bath for 10 min. To quantify the dissolved organic carbon (DOC) concentration of the HA solution, measurements were performed using an ELEMENTAR TOC carbon analyzer (LiquiTOC/TNb, Langenselbold, Germany).

Gram-negative *Pseudomonas putida* (Trevisan) Migula (ATCC^®^ 12633^TM^) (CCTCC AB 2014017, *P. p*) was supplied by the China Center for Type Culture Collection (Wuhan, Hubei, China). For initial activation, the *P. p* strain was inoculated into Luria–Bertani (LB) medium containing 10.0 g∙L^−1^ tryptone, 5.0 g∙L^−1^ yeast extract, and 5.0 g∙L^−1^ NaCl and incubated for 24–36 h at 37 °C. To obtain cells at the late-exponential growth phase, we cultivated the activated bacteria for 7–8 h at 28 °C and then performed expansion culture at a 1:100 ratio for an additional 18 h. Upon completion of cultivation, fresh biomass was collected by centrifugation at 5000 rpm for 12 min and washed three times with 0.01 mol∙L^−1^ NaNO_3_ electrolyte. The resulting cell pellets were resuspended in the electrolyte and stored at 4 °C. To determine biomass dry weight, a 1 mL aliquot of the *P. p* suspension was transferred into a pre-weighed 10 mL centrifuge tube and dried at 60 °C to constant weight. The biomass dry weight was calculated from the mass difference before and after drying and used to determine the amount of fresh bacterial suspension required for preparing mixtures at the desired Mont-to-bacteria dry-weight ratios. To preserve bacterial activity while minimizing the production of bacterial secretions, fresh *P. p* biomass was used for subsequent adsorption experiments within 12 h. Prior to these experiments, the obtained bacteria were finally suspended in 0.01 mol∙L^−1^ NaNO_3_ electrolyte.

Cd(NO_3_)_2_∙4H_2_O, Cu(NO_3_)_2_∙3H_2_O, tryptone, yeast extract, NaCl, NaNO_3_, HNO_3_, and NaOH were of analytical reagent grade and were obtained from Sinopharm Chemical Reagent Shenyang Co., Ltd. (Shenyang, Liaoning, China).

For preparation of the metal stock solutions, Cd(NO_3_)_2_∙4H_2_O and Cu(NO_3_)_2_∙3H_2_O were separately dissolved in Milli-Q water to obtain Cd(II) and Cu(II) solutions at 100 mg·L^−1^. Prior to the adsorption experiments, we diluted these stock solutions to the required concentrations. To establish the desired solution conditions, the initial pH was adjusted to 5.0 using 0.1 mol∙L^−1^ NaOH and HNO_3_, while 0.01 mol∙L^−1^ NaNO_3_ was introduced as the electrolyte solution to maintain a constant ionic strength. For pH determination, solution pH was measured using a pH meter (PHS-3C, Shanghai Instrument Scientific Instrument Co., Ltd., Shanghai, China).

### 2.2. Preparation of the Composites

#### 2.2.1. Preparation of Mineral-Based Composite

The mineral mixture was prepared by mixing montmorillonite and kaolinite at a mass ratio of 1:1 (Mont/Kao). Briefly, 2.0 g of montmorillonite and 2.0 g of kaolinite were mixed and suspended in 0.01 mol∙L^−1^ NaNO_3_ electrolyte. The suspension was dispersed using an ultrasonic bath for 10 min prior to use.

#### 2.2.2. Preparation of Mineral–Bacteria Binary Composites

The Mont/Kao-*P. p* binary composites were prepared by mixing the Mont/Kao mineral mixture with *P. p* suspensions at mineral-to-bacteria dry-weight ratios of 1:1, 10:1, and 100:1. The corresponding amounts of mineral and bacterial biomass were calculated based on their dry weights. The suspensions were shaken for 4 h at 28 °C and pH 5.0 to reach adsorption equilibrium between minerals and bacterial cells. At this pH condition, mont (PZC ≈ 2.5) and bacterial cells (PZC typically ranging from 2 to 4) are both negatively charged, leading to moderate electrostatic repulsion that may regulate, rather than prevent, mineral–bacteria interactions and promote stable interfacial association. No washing step was applied before the adsorption experiments.

#### 2.2.3. Preparation of Mineral–Humic Acid Binary Composites

The Mont/Kao-HA composites were prepared by reacting 2.0 g of Mont/Kao mixture with 200 mL of HA solution at initial concentrations of 100, 500, 800, 1200, and 1500 mg∙L^−1^. The suspension pH was adjusted to 5.0 using 0.1 mol·L^−1^ NaOH and HNO_3_, while 0.01 mol·L^−1^ NaNO_3_ was used as the background electrolyte to maintain a constant ionic strength. After shaking at 28 °C for 24 h, the suspension was centrifuged (8000 rpm, 12 min), washed three times with NaNO_3_ electrolyte, freeze-dried, ground, and sieved (<200 mesh). The residual HA concentration in the supernatant was determined using UV-vis spectroscopy at 314 nm to calculate HA loading on the mineral surface.

#### 2.2.4. Preparation of Mineral-HA-Bacteria Ternary Composites

The ternary Mont/Kao-HA-*P. p* composites were prepared by introducing *P. p* suspension dropwise into the preformed Mont/Kao-HA complex suspended in 0.01 mol∙L^−1^ NaNO_3_. The pH was maintained at 5.0 using 0.1 mol∙L^−1^ NaOH and HNO_3_. Two minerals: HA: bacteria dry-weight ratios (50:1:50 and 100:5:1) were selected to represent different organic matter and microbial activity conditions. The ratio 50:1:50 was selected to simulate a high microbial activity scenario, while 100:5:1 represents a system with moderate organic matter content typical of bulk agricultural soils. The suspensions were shaken for 24 h at 25 °C, followed by centrifugation and washing three times with 0.01 mol∙L^−1^ NaNO_3_. UV-vis analysis confirmed that free HA was negligible in the final suspensions (<1%), indicating effective HA immobilization. The obtained composites were finally resuspended in NaNO_3_ electrolyte for subsequent Cd(II) and Cu(II) adsorption experiments.

The detailed composition and preparation conditions of all mineral-based composites are summarized in Table 1.

### 2.3. Characterization

The soils and the composites, both before and after the adsorption of heavy metals, were characterized using SEM, XRD, and FTIR spectroscopy. All samples were freeze-dried and ground prior to analysis.

The surface topography and structure of samples were analyzed by SEM (HITACHI S-4800, Tokyo, Japan) using 20 kV acceleration voltage.

For soil samples, the 300-mesh deferrized soil was saturated with 1.0 mol∙L^−1^ KCl solution three times and washed once with distilled water to prepare a soil chromatography sheet for analysis. The composite suspensions, before and after heavy metal adsorption, were collected, quickly lyophilized (Labconco FreeZone, Kansas City, MO, USA) overnight, and stored in closed containers prior to analysis. XRD analysis was performed at 40 KV and 40 mA with CuKα radiation (*λ* = 1.5418 Å). Data were collected at room temperature over a 2*θ* range of 5° to 90° at a scanning rate of 10°·min^−1^.

To characterize changes in the functional groups associated with the adsorption reaction, FTIR analysis was performed on the minerals and their composites both before and after adsorption. Using KBr pellets, the spectra were recorded with a Thermal Fisher Nicolet 6700 FTIR spectrometer (Morristown, NJ, USA) over the range of 400~4000 cm with a resolution of 4 cm^−1^.

### 2.4. Adsorption Study

To evaluate Cd(II) and Cu(II) adsorption, batch experiments were conducted using Mont/Kao, *P. p*, Mont/Kao-HA, Mont/Kao-*P. p*, and Mont/Kao-HA-*P. p* suspensions under a fixed ionic strength of 0.01 mol∙L^−1^ NaNO_3_. Aliquots of each sorbent were transferred into a series of 50 mL polyethylene centrifuge tubes, followed by the addition of either Cd(II) or Cu(II) solution. For each tube, the final volume was brought to 25 mL with the electrolyte (0.01 mol∙L^−1^ NaNO_3_), yielding sorbent and Cd(II) or Cu(II) concentrations of 1.0 g∙L^−1^ and 8.0 mg∙L^−1^, respectively. Following pH adjustment, we equilibrated the mixtures for 4 h at room temperature (25 °C) and centrifuged them at 8000 rpm for 12 min. To separate suspended particles while retaining dissolved metal species, the resulting supernatant was passed through 0.45 μm nylon membrane filters (Shanghai Xinya Purification Equipment Co., Ltd., Shanghai, China), after which the concentration of unadsorbed heavy metal was determined using atomic absorption spectrometry (Varian SpectrAA-220; Agilent Technology, Inc., Santa Clara, CA, USA). For subsequent SEM, XRD and FTIR analysis, the resulting suspensions at pH 5.0 were quickly collected, freeze-dried, and stored in closed containers.

To quantify the equilibrium adsorption of Cu(II) or Cd(II), the adsorption capacity was calculated from the experimental data using the following equation:

(1) $$q_{e}=\frac{(C_{0}-C_{e})\cdot V}{m}$$

where *q*_e_ denotes the adsorption capacity at equilibrium (mg∙g^−1^), while *C*_0_ and *C*_e_ correspond to the initial and equilibrium concentrations of the heavy metal (mg∙L^−1^), respectively. In this equation, *V* represents the solution volume (L), and *m* represents the mass of the sorbent (g).

To further evaluate the adsorption behavior, experiments were conducted by varying the initial heavy metal concentration, solution pH (range of 3–9), and temperature, as well as by examining the competitive adsorption of Cu(II) and Cd(II) on the composites.

To investigate the adsorption isotherms, experiments were performed with initial Cd(II) or Cu(II) concentrations ranging from 2 to 200 mg∙L^−1^ in 0.01 mol∙L^−1^ NaNO_3_ background electrolyte solutions. The experiments were carried out in 50 mL polyethylene centrifuge tubes at a pH of 5.0 with a sorbent dosage of 1.0 g∙L^−1^. To establish the adsorption equilibrium under different temperature conditions, we elliptically shook the mixtures at 180 rpm for 24 h on a thermostat water bath shaker maintained at 25, 30, 35 and 45 °C. Upon completion of shaking, the suspensions were centrifuged at 4000 rpm for 10 min, after which the residual concentrations of heavy metals in the solution were measured. All treatments were conducted in triplicate. For kinetic evaluation, the adsorption kinetics were analyzed using pseudo-first-order and pseudo-second-order models:

(2) $$q_{t}=q_{e}(1-e^{-k_{1}t})$$

(3) $$q_{t}=\frac{k_{2}q_{e}^{2}t}{1+k_{2}q_{e}t}$$

In these kinetic models, *q*_t_ denotes the adsorption capacity (mg·g^−1^) at time *t* (min), whereas *k*_1_ (min^−1^) and *k*_2_ (g·mg^−1^·min^−1^) represent the corresponding rate constants. To characterize the equilibrium adsorption behavior, the adsorption isotherm data were fitted using both the Langmuir and Freundlich models. Specifically, the Langmuir adsorption model was expressed as follows:

(4) $$q_{e}=\frac{q_{\max}bC_{e}}{1+bC_{e}}$$

The Freundlich adsorption model was applied:

(5) $$q_{e}=k_{f}C_{e}^{1/n}$$

For the adsorption isotherm parameters, *q*_max_ represents the theoretical maximum capacity (mg·g^−1^), while *b* (L·mg^−1^) and *k*_f_ (mg·g^−1^(L·mg^−1^)^1/n^) denote the corresponding affinity constants; n describes the heterogeneity. To further evaluate the thermodynamic characteristics of the adsorption process, the thermodynamic parameters Δ*G*°, Δ*H*°, and Δ*S*° were determined using the Van’t Hoff equations:

(6) $$∆G^{{}^{\circ}}=-\mathrm{RT}\ln K$$

(7) $$∆G^{{}^{\circ}}=∆H^{{}^{\circ}}-T∆S^{{}^{\circ}}$$

(8) $$\ln K=\frac{∆S^{{}^{\circ}}}{R}-\frac{∆H^{{}^{\circ}}}{\mathrm{RT}}$$

(9) $$K=\frac{q_{e}}{C_{e}}$$

(10) $$\ln\left(\frac{q_{e}}{C_{e}}\right)=\alpha q_{e}+\ln K$$

In these thermodynamic equations, *R* denotes the universal gas constant (8.314 J·mol^−1·^K^−1^), and T represents the absolute temperature (K). The solid–liquid distribution coefficient *K* (L·g^−1^) was obtained from the intercept of the plot of ln(*q*_e_/*C*_e_) versus *q*_e_.

To further investigate the adsorption behavior, an additional set of batch adsorption experiments was conducted at 28 °C under a fixed ionic strength of 0.01 mol∙L^−1^ NaNO_3_. For preparation of the adsorption systems, each sorbent suspension (1 mL, 10 g∙L^−1^, dry-weight basis) was transferred into a series of 20 mL centrifuge tubes, followed by the addition of different volumes of Cd(II) solution. We adjusted the final volume of each tube to 10 mL using 0.01 mol∙L^−1^ NaNO_3_, resulting in Cd(II) concentrations ranging from 0 to 0.5 mmol∙L^−1^. To achieve adsorption equilibrium, the centrifuge tubes were shaken at 40 g for 12 h, while the pH of each suspension was maintained at 5.0 through minor additions of 0.1 mol·L^−1^ HNO_3_ or 0.1 mol·L^−1^ KOH solution. Preliminary kinetics experiments confirmed that Cd adsorption reached equilibrium within 12 h. Upon completion of equilibration, the final suspensions were centrifuged at 8000 g and filtered through a 0.45 μm membrane. For these experiments, adsorption was evaluated as a function of initial Cu(II)/Cd(II) concentration, solution pH, sorbent dosage, and ionic strength. Prior to use, all glassware and bottles were pretreated.

### 2.5. Statistical Analysis

All experiments were independently performed in triplicate, and the data are presented as the mean ± standard deviation (SD). Statistical analyses were conducted using SPSS 18.0. A *p*-value < 0.05 was considered statistically significant. Different lowercase letters indicate significant differences among groups (*p* < 0.05).

## 3. Results and Discussion

### 3.1. Analysis and Characterization of Soil Minerals in Vegetable Greenhouses of Liaoning Province

The physicochemical characterization of clay minerals from agricultural soils in Liaoning Province, conducted via SEM, XRD, and FTIR, revealed a mineralogical composition predominantly comprising montmorillonite and kaolinite, alongside quartz and mica. These findings are consistent with prior mineralogical analyses of Haplic Cambisols and Dystric Cambisols in Northeast China [33].

**Figure 1:**
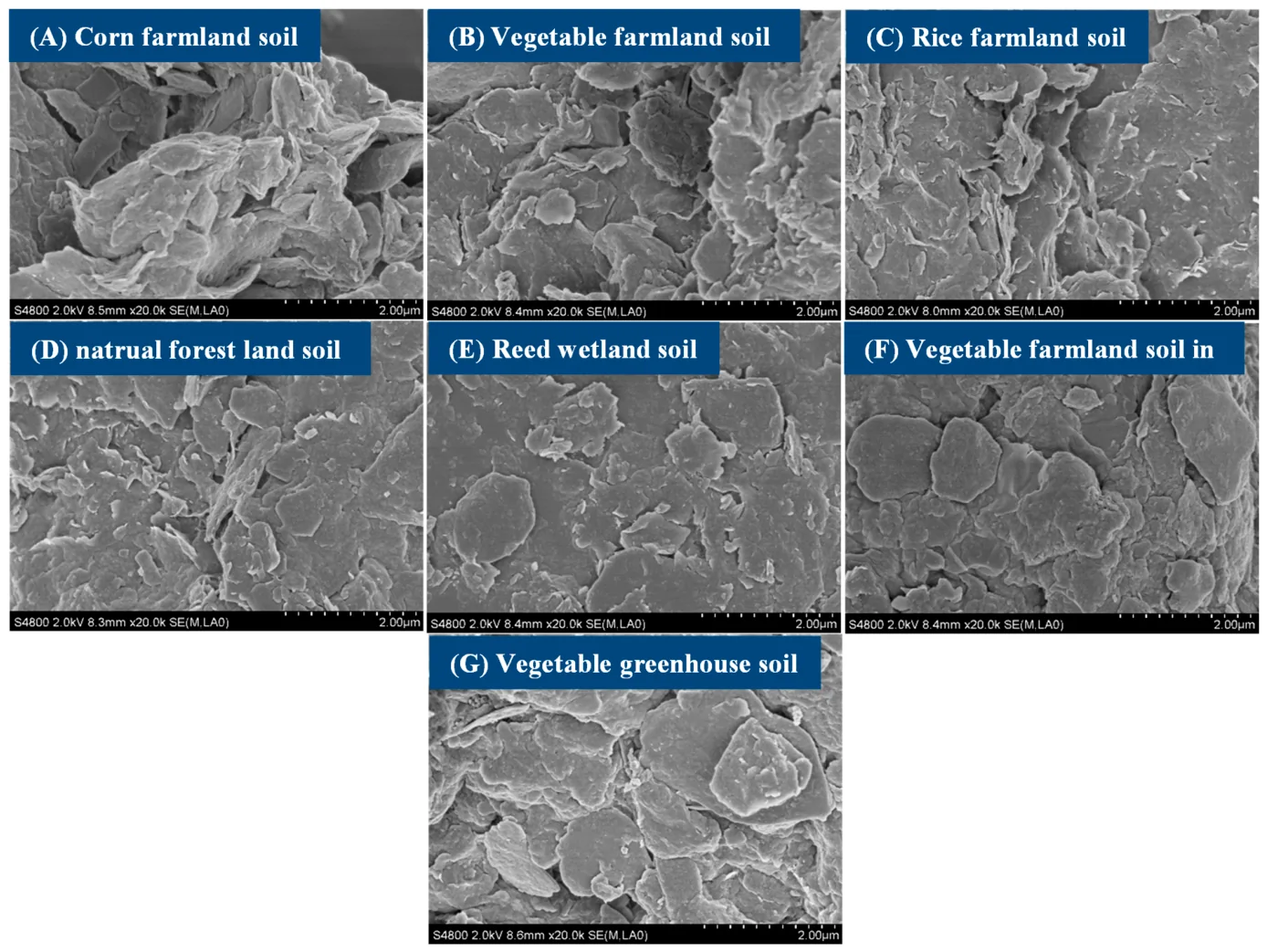
SEM images of soils from different land-use types: (**A**) corn farmland soil; (**B**) vegetable farmland soil; (**C**) rice farmland soil; (**D**) natural forestland soil; (**E**) reed wetland soil; (**F**) vegetable farmland soil in experimental base; (**G**) vegetable greenhouse soil.

SEM observations revealed distinct differences in soil microstructures among different land-use types (Figure 1). Soils from natural forestland and wetland exhibited relatively complex and heterogeneous structures with abundant pore spaces, likely associated with greater organic matter accumulation and biological activity. In contrast, agricultural soils displayed more compacted structures as a result of intensive cultivation. Greenhouse vegetable soils showed pronounced aggregation and surface coverage, suggesting that long-term intensive management altered soil particle arrangement and microhabitat characteristics.

**Figure 2:**
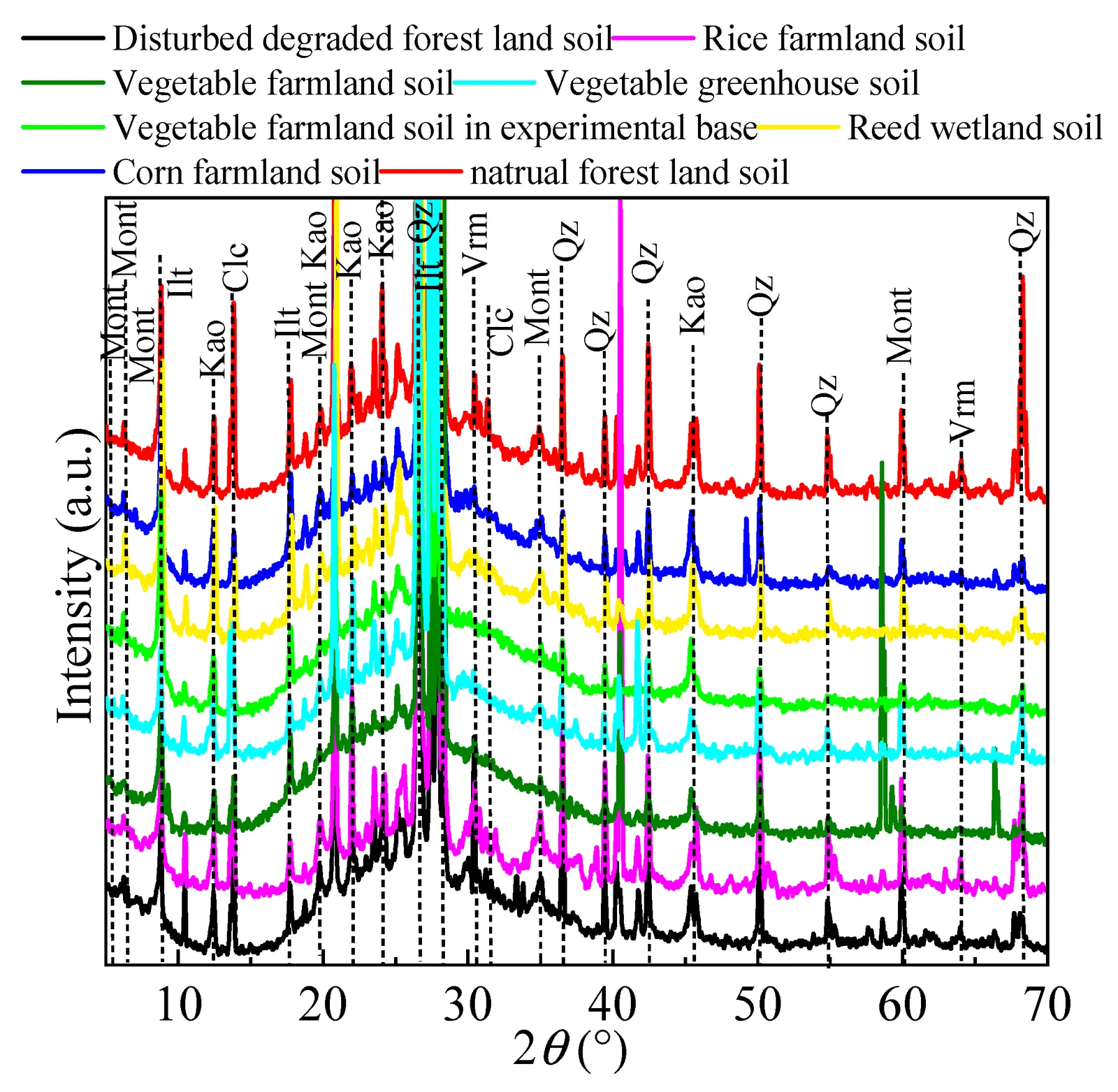
XRD patterns of forest land and vegetable farmland soils.

The XRD patterns (Figure 2) revealed characteristic reflections attributable to quartz (Qz) at 3.34 Å and 1.82 Å, montmorillonite (Mont) at 14.9 Å (JCPDS 00-002-0014), and kaolinite (Kao) at 7.15 Å and 4.35 Å (JCPDS 75-0938), confirming the occurrence of these mineral phases in the soil samples. Minor phases of illite (Ilt), vermiculite (Vrm), and chlorite (Clc) were also identified.

**Figure 3:**
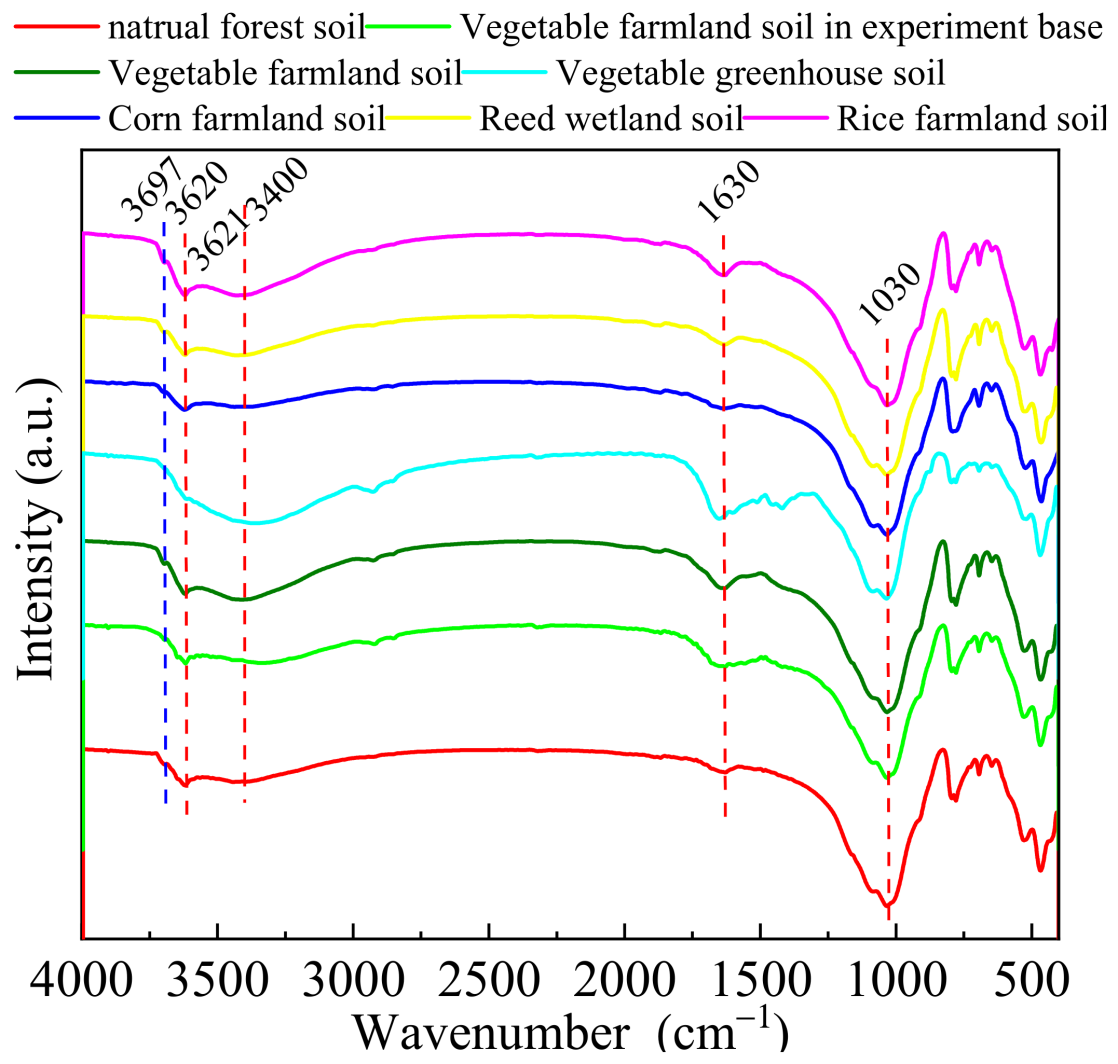
FTIR patterns of forest land and vegetable farmland soils.

The FTIR spectra (Figure 3) further corroborated this mineral assemblage identified by XRD. Specifically, the broad bands centered at approximately 3400 cm^−1^ and 1630 cm^−1^, together with the feature near 3620 cm^−1^, correspond to O-H stretching vibrations associated with interlayer water and structural hydroxyl groups in montmorillonite, as well as the H-O-H bending vibration of adsorbed/interlayer water. Simultaneously, the sharp peak at 3697 cm^−1^ and the distinct peak at 3621 cm^−1^ represent characteristic vibrational signatures of kaolinite, arising from the stretching vibrations of surface hydroxyl groups and inner Al-OH groups within the alumina octahedral sheet, respectively [33,34]. Samples B, E and G exhibited more prominent kaolinite features, indicating a relatively higher abundance of kaolinite in these samples.

While this characterization confirms the presence of these two dominant clay minerals, it also highlights a key limitation: natural soils are complex mixtures containing minor phases (mica, palygorskite, Fe/Al oxides, and organic matter) that can collectively influence metal adsorption via synergistic or competitive effects [23]. The surface charge heterogeneity induced by permanent (montmorillonite basal planes) and variable charge sites (kaolinite edge hydroxyls and Fe/Al oxide surfaces) creates a complex electrostatic landscape that is difficult to replicate in simplified model systems. Therefore, to isolate the fundamental mechanisms governing heavy metal adsorption, this study strategically selected Mont and Kao as representative, pure-phase layered silicates. A binary Mont/Kao composite (1:1 mass ratio) was then prepared to serve as a simplified yet representative model for the soil clay fraction. This approach allows for a controlled investigation of the adsorption and binding mechanisms of Cd(II) and Cu(II) onto the montmorillonite-humic acid-bacteria ternary complex, minimizing the confounding effects of other soil components. By systematically building complexity from single minerals to binary and ternary composites, while considering the effects of mineral composition and interactions, this work aims to provide fundamental theoretical support for predicting the fate of heavy metals in real soil systems. Future studies should further incorporate variations in contaminant concentrations, organic matter contents, and mineral proportions to better capture the complexity of natural soils and guide the development of clay-based remediation materials through interlayer modification or surface functionalization [24].

### 3.2. Adsorption Experiment

#### 3.2.1. Effect of the Initial Metal Solution pH and Composite Type

Solution pH is a master variable governing the adsorption of heavy metals, critically influencing the speciation of metal ions, the protonation–deprotonation equilibria of surface hydroxyl groups on minerals and HA, and the surface charge, isoelectric point, and hydrophobicity of bacterial cells [29,35]. The interplay of these factors dictates the adsorption behaviors of Cu(II) and Cd(II) onto the multi-component composites.

**Figure 4:**
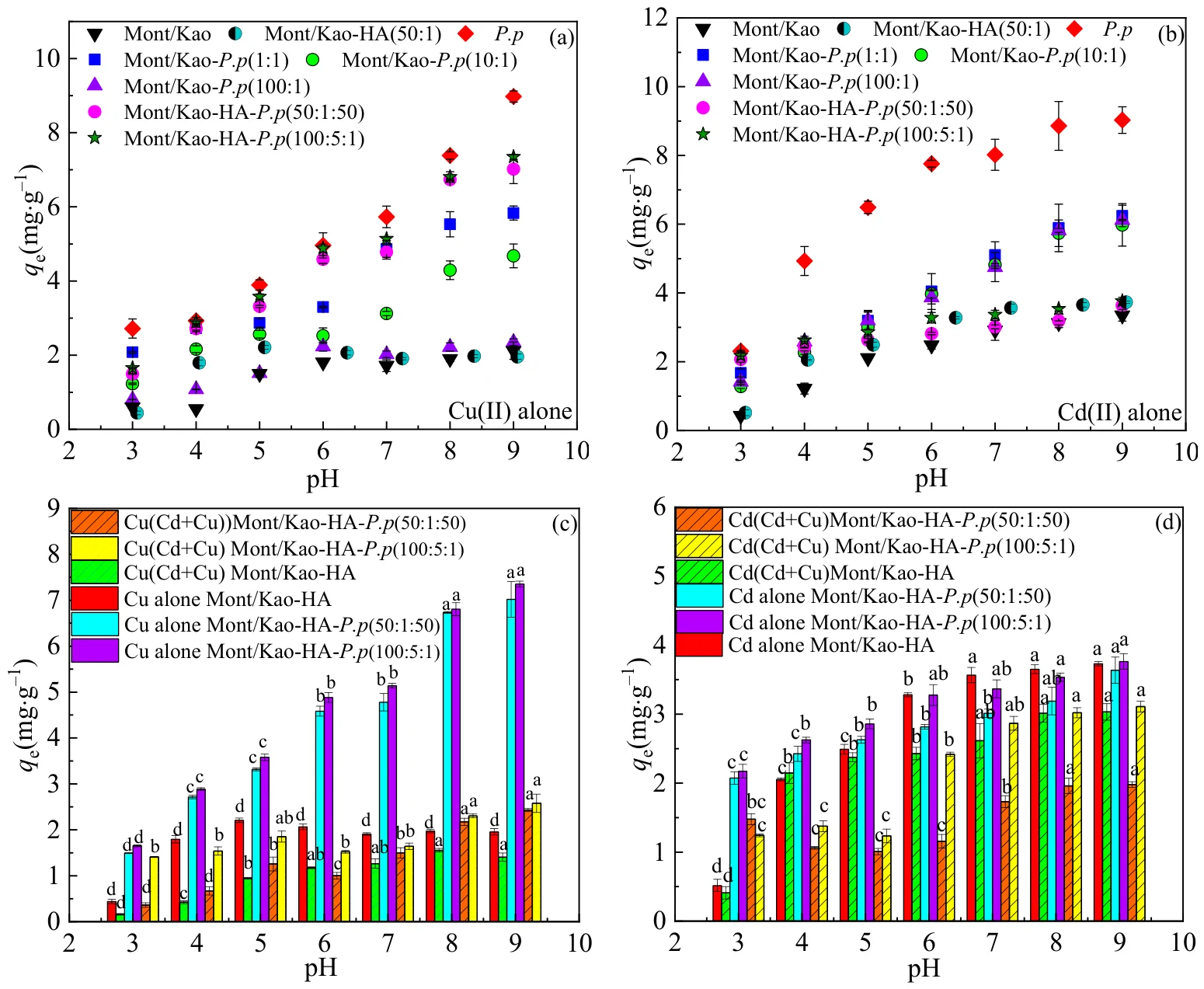
Effect of pH on Cu(II) and Cd(II) adsorption onto composites: (**a**) pH and adsorption capacity (*q_e_*) in monometallic Cu(II) systems; (**b**) pH and *q_e_* in the monometallic Cd(II) system; (**c**,**d**) competitive adsorption in the Cu-Cd binary system: pH versus *q_e_* for Cu(II) (**c**) and Cd(II) (**d**). (Initial concentrations: *C*_Cu0_: 7.5 mg∙L^−1^; *C*_Cd0_: 9.4 mg∙L^−1^, adsorbent dosage: 1.0 g∙L^−1^; ionic strength: 0.01 mol∙L^−1^; *T*: 25 °C.) Data are presented as mean ± SD (*n* = 3). Different lowercase letters indicate significant differences among treatments (*p* < 0.05).

As illustrated in Figure 4a,b, the adsorption capacity of both Cu(II) and Cd(II) onto the various sorbents in single-metal systems increased progressively with pH, with a marked decrease under highly acidic conditions (pH < 4). While this general trend was similar, the underlying driving forces differed due to their disparate hydrolysis constants and affinities for specific functional groups. In the pH range of 3–6, both metals exist predominantly as free divalent cations, and ion exchange with permanent charge sites on clay minerals constitutes a primary process [23,36].

The point of zero charge (pH_pzc_) for montmorillonite and kaolinite is approximately 2.3 and 2.6, respectively, so the progressively negative net charge on mineral surfaces at pH > 3 enhances electrostatic attraction for both cations. However, for Cu(II), hydrolysis becomes significant at pH > 6.0, generating species like Cu(OH)^+^ which are strongly adsorbed via inner-sphere complexation, leading to a synergistic effect with ion exchange. In contrast, Cd(II) remains predominantly as the free Cd(II) ion well into the alkaline range [35], making its adsorption more dependent on the deprotonation of specific functional groups. Mechanistically, Cd(II) adsorption proceeds through distinct stages: Outer-sphere complexation with mineral permanent charge sites at pH < 4.5, transitioning to inner-sphere complexation with carboxyl groups at pH 4.5–5.5, and finally to dominant binding with phosphoryl groups at pH > 5.5. This shift explains why Cd(II) adsorption, while quantitatively lower, continued to increase steadily across the pH 4–8 range.

The incorporation of humic acid and bacteria further modulated these pH-dependent trends. Under acidic conditions, the protonation of carboxyl and hydroxyl groups on HA increases surface hydrophobicity, hindering cation adsorption, while deprotonation at pH > 7 enhances metal complexation [29,37]. The bacterial cell wall structure played a decisive role. In Gram-negative *P. p*, the high density of anionic groups is primarily associated with the outer membrane (lipopolysaccharide layer), rather than the peptidoglycan layer [38]. The overall adsorption hierarchy in single-metal systems was consistent: *P. p* > Mont/Kao-HA-*P. p* > Mont/Kao-*P. p* > Mont/Kao-HA > Mont/Kao, confirming that systems with higher organic (especially bacterial) content exhibited superior adsorption capacity. This is attributed to the non-occlusive, loose association formed between minerals and bacteria via hydrophobic interactions, which preserves the binding sites on both components [39,40].

As shown in Figure 4c,d, in the Cu-Cd binary system, the presence of Cd(II) significantly suppressed Cu(II) adsorption across the entire pH range, an effect most pronounced on composites with high bacterial content such as *P. p*. Conversely, the negative impact of Cu(II) on Cd(II) adsorption was far less pronounced. Cu(II), as a borderline Lewis acid with a strong affinity for both carboxyl and phosphoryl groups on bacterial surfaces, faces intense competition for these limited, high-affinity sites from Cd(II). However, Cd(II) possesses a critical advantage: as established in the literature, it preferentially displaces Ca^2+^ from the interlayer of Ca-montmorillonite through ion exchange, whereas Cu(II) preferentially displaces Na^+^. Since the clay minerals in our composites likely have a more abundant pool of Ca^2+^ available for exchange, Cd(II) can access this substantial “reserved” pool of mineral sites that Cu(II) cannot efficiently utilize. This dual binding pathway-via both bacterial organic sites and mineral interlayer sites-makes Cd(II) a formidable competitor.

The relatively stable adsorption trends across the investigated pH range indicate that adsorption was controlled by multiple complementary mechanisms rather than a single pH-sensitive process. The coexistence of mineral exchange sites, HA functional groups, and bacterial surface ligands likely buffered the effect of pH variation on overall metal retention.

#### 3.2.2. Effect of Initial Cd(II)/Cu(II) Concentration on Adsorption

The adsorption behavior of heavy metals is strongly influenced by solution pH, metal concentration, and sorbent properties.

**Figure 5:**
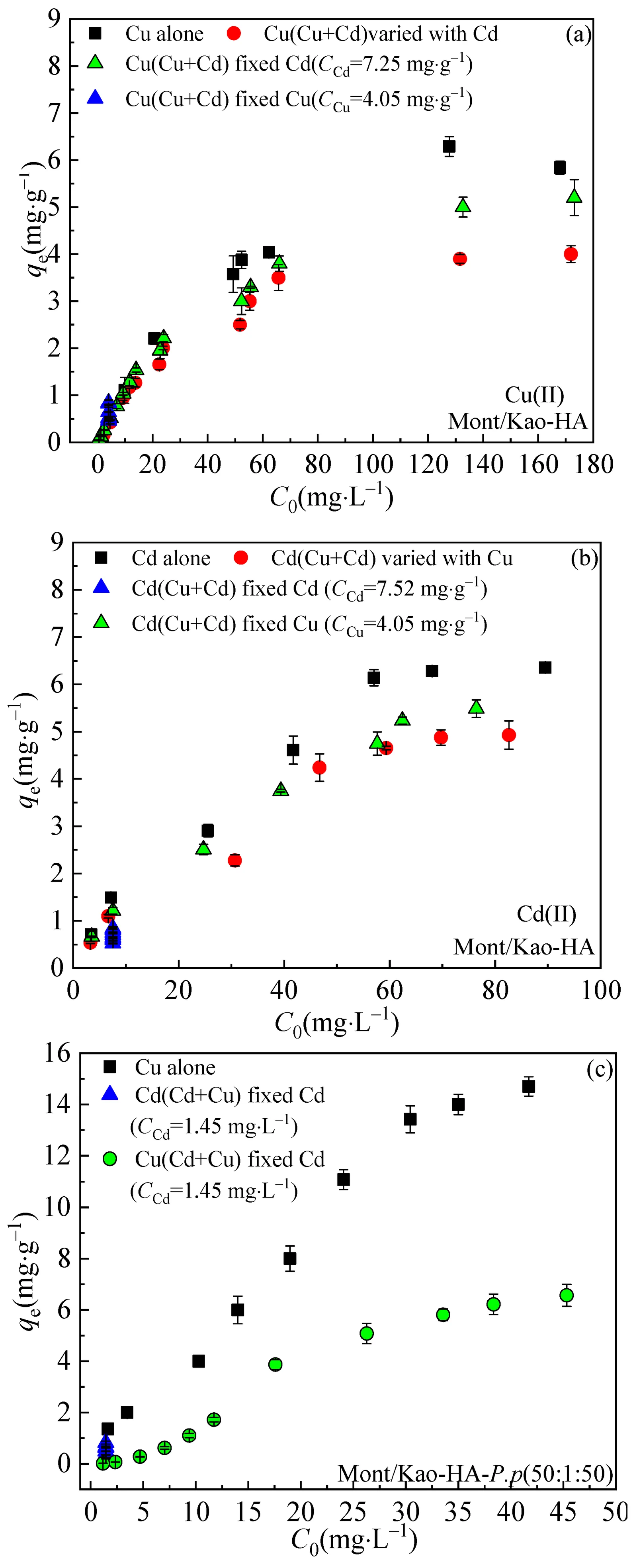

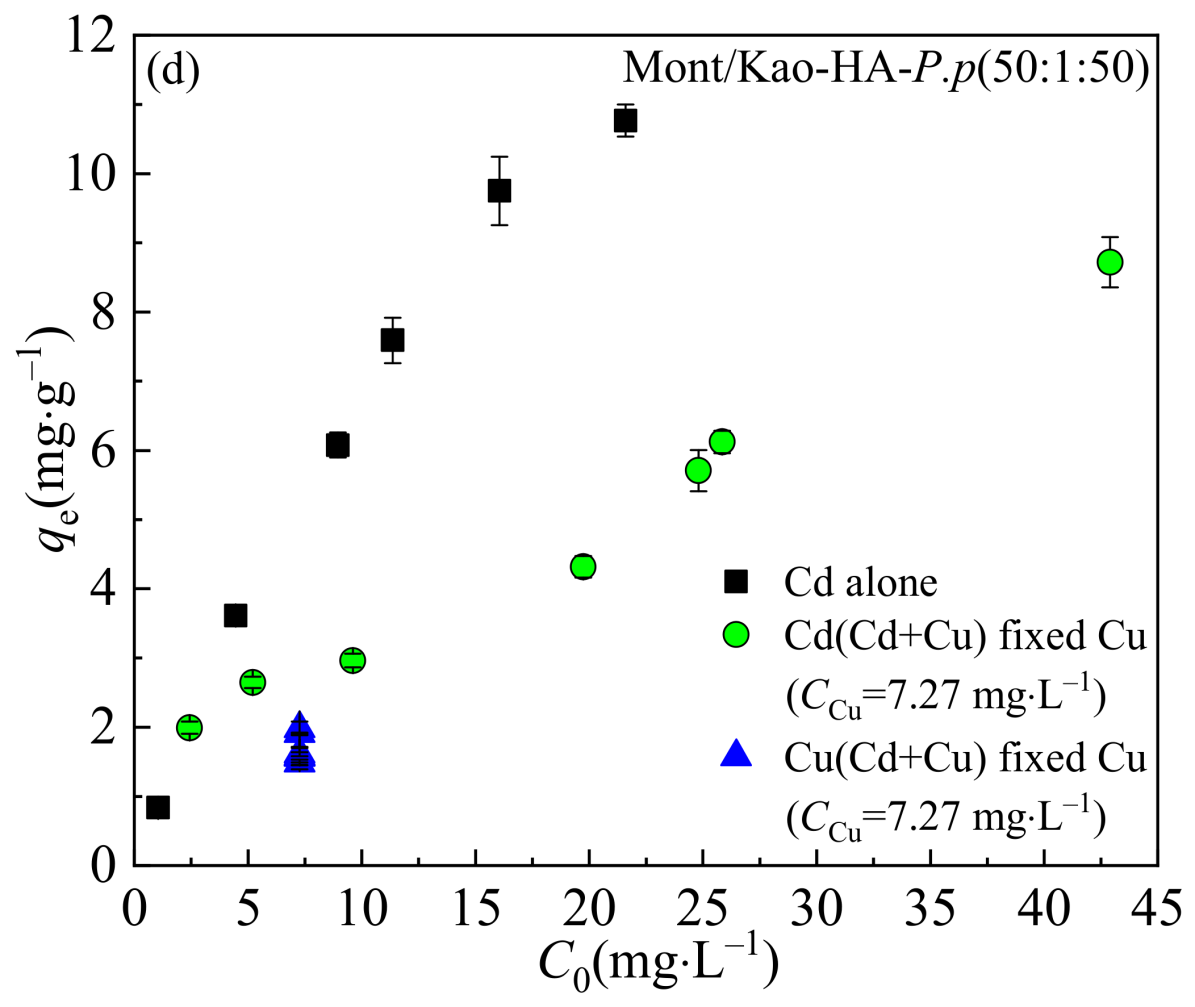
Effect of initial concentration on Cu (II) and Cd (II) adsorption: (**a**) Cu(II) adsorption; (**b**) Cd(II) adsorption; (**c**) competitive adsorption of Cu(II); (**d**) competitive adsorption of Cd(II). (Adsorbent dosage: 1.0 g∙L^−1^; ionic strength 0.01 mol∙L^−1^; pH 5.0; *T* 25 °C.) (Data are presented as mean ± SD (*n* = 3).

As shown in Figure 5, with the sorbent concentration fixed at 1.0 g·L^−1^, the adsorption capacities of Cd(II) and Cu(II) increased with initial metal concentration for all composites, owing to the increased concentration gradient and mass-transfer driving force. At low concentrations, adsorption mainly occurred at high-affinity sites, resulting in similar behavior across the composites. However, as metal loading increased, differences among the composites became more pronounced. Mont/Kao-*P. p* showed the greatest increase in adsorption capacity, whereas Mont/Kao-HA gradually approached saturation, reflecting differences in adsorption-site density and accessibility. Although sorbent dosage was not varied in this study, it is an important parameter in practical remediation. Increasing composite concentration is expected to enhance overall metal removal by providing additional adsorption sites; however, excessive dosage may reduce adsorption capacity per unit mass because of particle aggregation and reduced accessibility of active sites.

These differences reflect the distinct binding domains contributed by the individual components. Mineral surfaces mainly provide ion-exchange sites, whereas HA introduces additional carboxyl and phenolic groups for metal complexation [20,41]. At low HA loading, these functional groups effectively enhance metal retention by providing additional complexation sites. However, at high metal concentrations, competition among metal ions and occupation of HA functional groups progressively reduce the availability of binding sites, leading to gradual saturation. Bacterial biomass provides abundant functional groups, including carboxyl, phosphoryl, amino, and hydroxyl groups [40], creating diverse binding environments that sustain adsorption at higher metal concentrations. Therefore, bacterial modification preferentially enhanced Cu(II) adsorption because of its stronger affinity for oxygen-containing ligands and tendency to form inner-sphere complexes, whereas Cd(II) can also bind to permanent-charge sites on minerals via ion exchange.

A critical distinction between the two metals was also evident: the performance gap between bacterial and non-bacterial composites was substantially greater for Cu(II) than for Cd(II). Although the contrasting coordination preferences of Cu(II) and Cd(II) are well established, their consequences for metal partitioning between bacterial and mineral domains in such composite systems cannot be inferred from HSAB theory alone. Our comparative experiments demonstrate that the incorporation of bacteria contributes more strongly to Cu(II) immobilization. This behavior is consistent with the greater tendency of Cu(II), a borderline Lewis acid, to form inner-sphere complexes with oxygen-donor functional groups, such as carboxyl and phosphoryl groups, abundant on bacterial surfaces. Cd(II) can also interact strongly with these functional groups, but it retains a greater contribution from ion-exchange interactions with permanent-charge sites on minerals. Consequently, Cd(II) immobilization is comparatively less dependent on the bacterial organic-rich domains. These results therefore experimentally quantify how established differences in metal coordination chemistry translate into distinct contributions of bacterial and mineral components to metal immobilization.

In multi-metal systems, Cd(II) and Cu(II) compete for limited adsorption sites rather than behaving independently. In contrast, Cd(II) can retain a greater capacity for outer-sphere ion exchange with permanent charge sites on minerals, owing to its ability to compete with major cations such as Ca(II). Meanwhile, Cd(II) may also interact with organic-rich domains through specific surface complexation, although its retention is not solely dependent on organic matter. These results demonstrate that metal retention in mineral–organic–microbial composites depends not only on adsorption capacity but also on the selective partitioning of metals among different interfacial binding sites.

### 3.3. Adsorption Isotherms

#### 3.3.1. Adsorption Efficiency Cu and Cd Adsorption Isotherms in Single Systems

**Figure 6:**
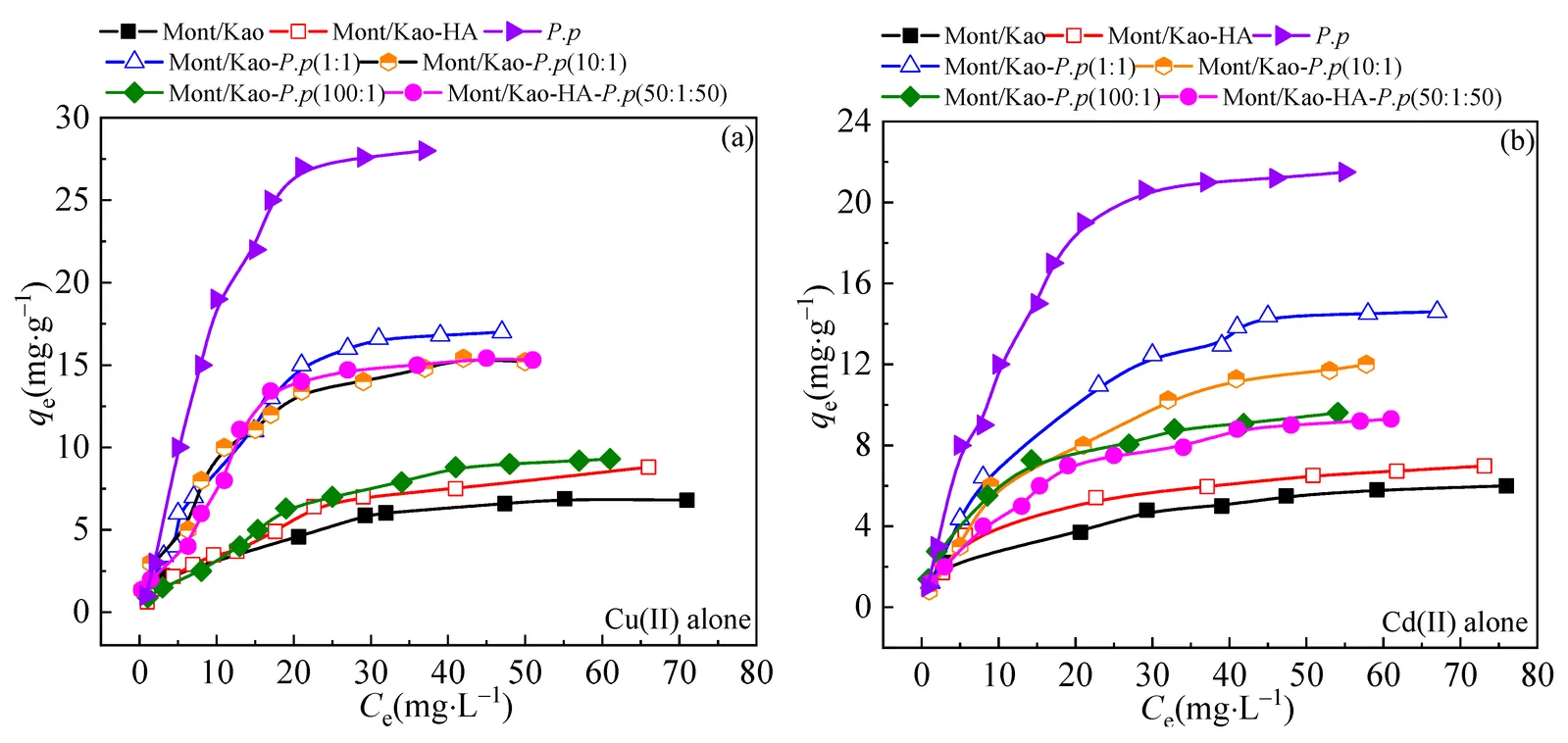
Adsorption isotherms of Cu(II) (**a**) and Cd(II) (**b**) on mineral, HA and bacteria composites. (*T*: 25 °C, adsorbent dosage: 1.0 g∙L^−1^, ionic strength: 0.01 mol∙L^−1^, pH: 5.0).

**Figure 7:**
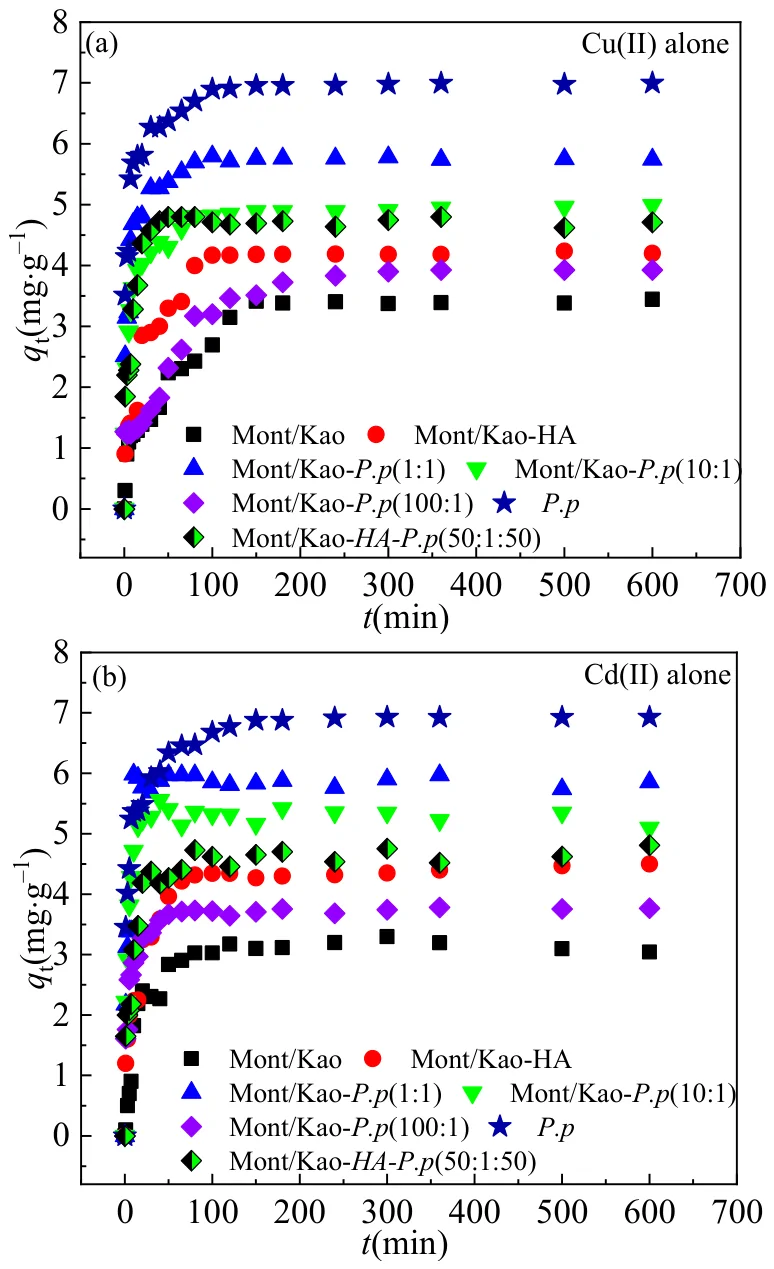
Adsorption kinetics of (**a**) Cu(II) and (**b**) Cd(II) onto Mont/Kao, HA and bacteria composites. (*C*_0_: 20.0 mg∙L^−1^, *T*: 28 °C, adsorbent dosage 2.0 g∙L^−1^, ionic strength: 0.01 mol∙L^−1^, pH 5.0).

**Figure 8:**
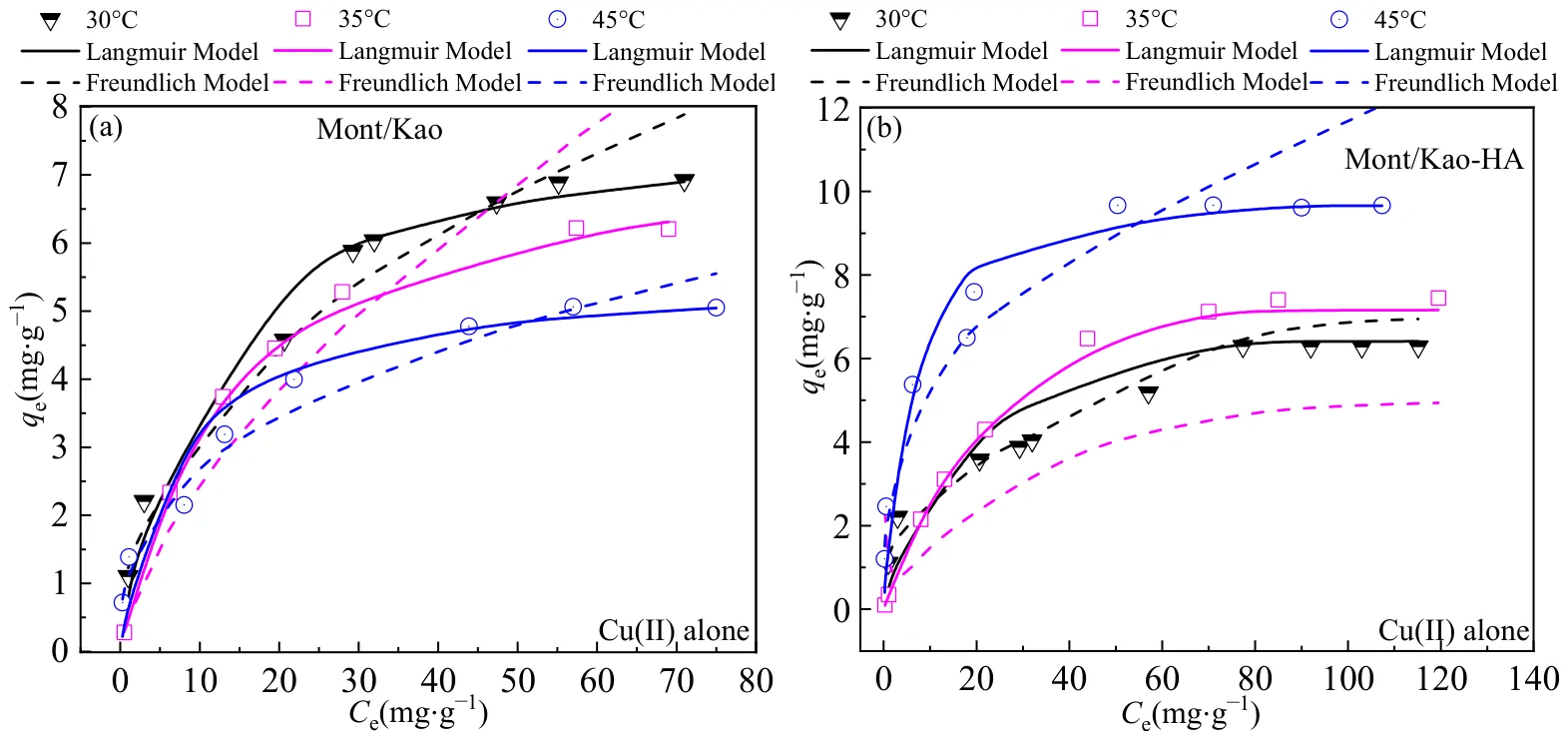

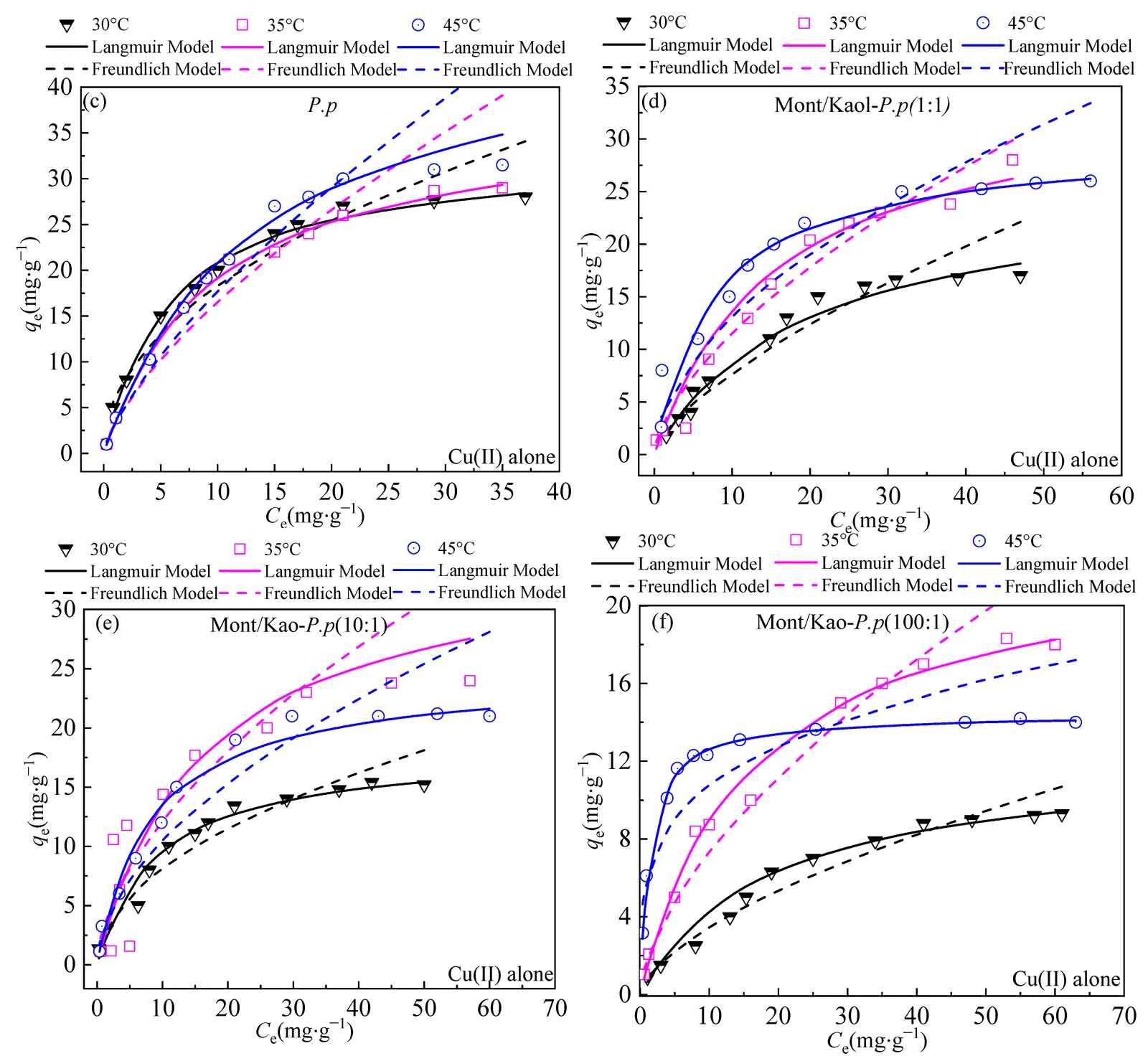

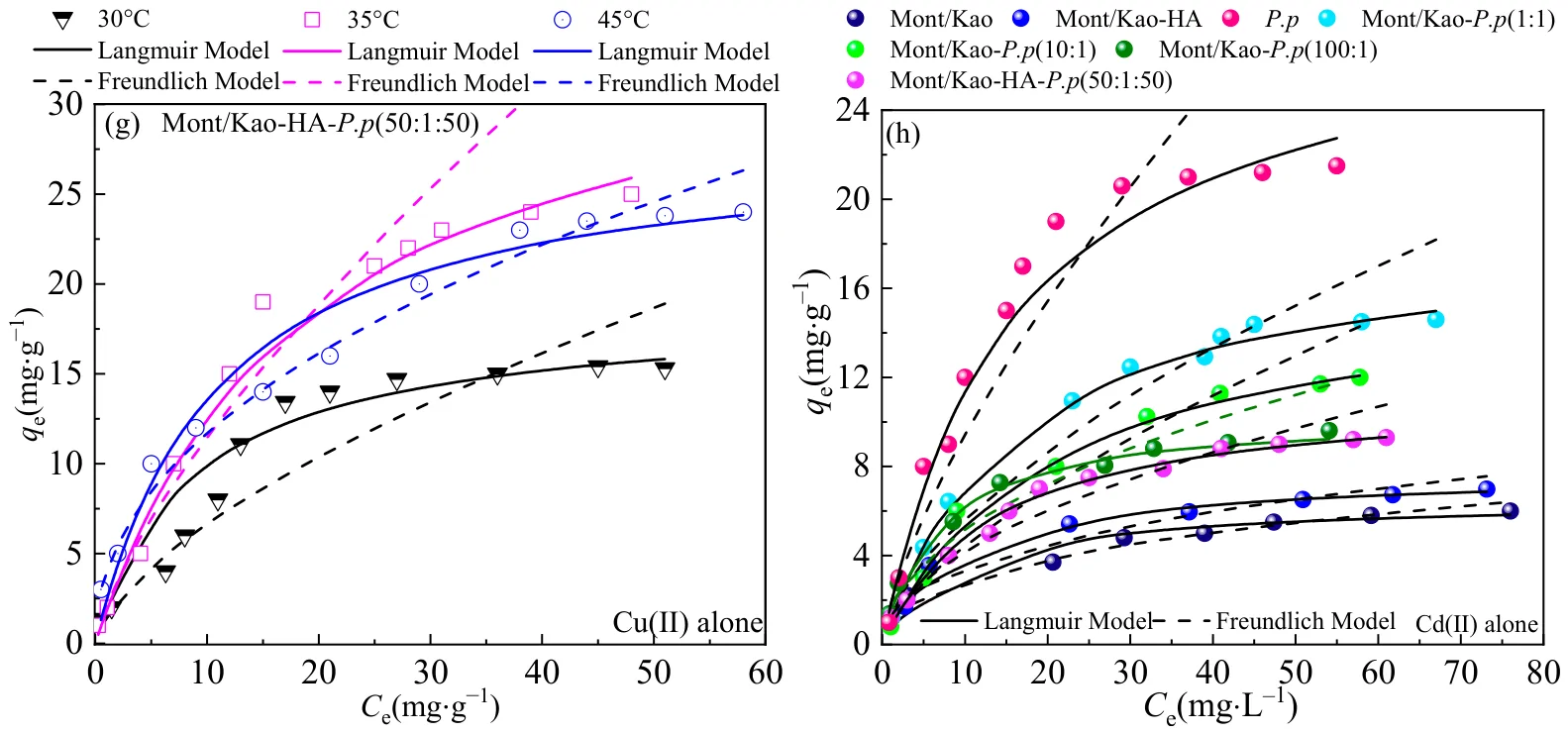
Cu(II) and Cd(II) adsorption isotherms and model fitting on pristine and composites: Equilibrium adsorption behavior of Cu(II) on (**a**) Mont/Kao, (**b**) Mont/Kao-HA, (**c**) *P. p*, (**d**) Mont/Kao-*P. p* (1:1), (**e**) Mont/Kao-*P. p* (10:1), (**f**) Mont/Kao-*P. p* (100:1), and (**g**) Mont/Kao-HA-*P. p* (50:1); (**h**) Comparative summary of Cd(II) adsorption isotherms at 25 °C. (Adsorbent dosage: 1.0 g∙L^−1^; pH: 5.0; ionic strength: 0.01 mol∙L^−1^; *t*: 48 h).

The equilibrium adsorption and kinetic behavior of Cu(II) and Cd(II) on clay minerals, *P. p*, humic acid, and their composites are presented in Figure 6, Figure 7 and Figure 8, with model parameters summarized in Table 1 and Table 2. Metal retention was controlled by the abundance, accessibility, and spatial distribution of chemically distinct binding sites.

As shown in Figure 6, the equilibrium adsorption capacities of both metals generally followed the order *P. p* > Mont/Kao-*P. p* composites > Mont/Kao-HA-*P. p* > Mont/Kao-HA > Mont/Kao. Among the mineral–bacterium composites, adsorption capacity decreased as the proportion of mineral increased, following the sequence Mont/Kao-*P. p* (1:1) > Mont/Kao-*P. p* (10:1) > Mont/Kao-*P. p* (100:1). The high adsorption capacity of *P. p* can be attributed to the abundance of carboxyl, phosphoryl, and hydroxyl groups on the bacterial cell surface, which provide effective coordination sites for divalent metal ions.

The adsorption capacities of the binary Mont/Kao-*P. p* and Mont/Kao-HA composites generally fell between those of their corresponding individual components, indicating that incorporation of bacterial biomass or humic acid modified the overall metal adsorption behavior of the mineral-based systems. In the mineral–bacterium composites, electrostatic repulsion between negatively charged clay particles and bacterial cells may limit close aggregation, resulting in a less compact and more dispersed association between the two components. This configuration may preserve the accessibility of adsorption sites on both components and provide a broader range of potential binding domains than the mineral component alone.

In contrast, the ternary Mont/Kao-HA-*P. p* composite exhibited a lower adsorption capacity than the corresponding Mont/Kao-*P. p* binary composite, despite all composite systems being compared at the same total adsorbent dosage and initial metal concentrations. This decrease suggests a non-additive response arising from interactions among the three components, which is attributed to partial masking or blocking of mineral- and bacteria-associated adsorption sites by HA and competition among interfacial binding domains. This behavior was attributed to the simultaneous occurrence of site enrichment and site masking. Additional carboxylic and phenolic groups were introduced by HA, and metal bridging among minerals, bacterial cells, and organic matter may thereby have been promoted [10,42]. However, high-affinity functional groups, particularly those on the bacterial envelope, may also have been sterically or electrostatically shielded by HA adsorbed on bacterial surfaces and mineral edges, while metal diffusion into internal interfacial regions may have been restricted. Consequently, the adsorption performance of the ternary composite was governed not simply by the total amount of reactive material, but by the spatial arrangement and effective accessibility of the available binding sites.

A consistent metal-specific difference was also observed, with a higher adsorption capacity generally being obtained for Cu(II) than for Cd(II). This selectivity was associated with the combined effects of hydrated ionic radius and coordination affinity. Owing to its smaller hydrated radius, a closer approach of Cu(II) to mineral surfaces was facilitated, and access to montmorillonite interlayers and mineral-edge sites may have been enhanced. In addition, partial dehydration could be more readily achieved by Cu(II), allowing stable inner-sphere complexes to be formed with bacterial carboxylate and phosphate groups, HA functional groups, and mineral-edge hydroxyls. By contrast, because of its larger hydration shell and weaker affinity for oxygen- and phosphorus-containing ligands, Cd(II) was retained to a greater extent through ion exchange, electrostatic attraction, and comparatively nonspecific surface interactions.

The higher uptake of Cu(II) in the single-metal systems therefore could not be attributed to ionic size alone. Instead, it was ascribed to greater access to interlayer and edge sites, stronger complexation with organic functional groups, and preferential occupation of high-affinity adsorption domains. Overall, adsorption efficiency was controlled by the balance among site abundance, site accessibility, interfacial organization, and metal-specific binding chemistry.

Further insight into the adsorption mechanisms was provided by the kinetic profiles in Figure 7. For all composites, HM adsorption was characterized by a rapid initial stage within 0–40 min, followed by a progressively slower approach to equilibrium. As shown by the kinetic fitting results in Table 2, the pseudo-second-order model provided the best description of all systems, whereas substantially poorer fits were obtained with the pseudo-first-order model. This pattern was interpreted as evidence that the adsorption rate was predominantly controlled by chemically mediated interactions involving electron sharing or exchange between the metal ions and surface functional groups, resulting in the formation of inner-sphere complexes [43]. Such behavior was observed not only for the bacterial and organic-rich composites, but also for the unmodified mineral, indicating that Cu(II) uptake by Mont/Kao was governed by chemical interactions rather than by physical diffusion alone.

The HM adsorption rates followed the order Mont/Kao-HA-*P. p* > Mont/Kao-*P. p* (1:1) > *P. p* > Mont/Kao-*P. p* (10:1) > Mont/Kao-HA > Mont/Kao > Mont/Kao-*P. p* (100:1). A higher adsorption rate was obtained for Mont/Kao-HA than for the bare mineral, indicating that Cu(II) and Cd(II) uptake was promoted by HA incorporation. This enhancement has been attributed to the formation of cationic bridges between HA functional groups and mineral surfaces [16,42].

More notably, the highest adsorption rate was observed for the ternary Mont/Kao-HA-*P. p* composite, although its equilibrium capacity was lower than that of the Mont/Kao-*P. p* binary composite. This apparent kinetic-capacity trade-off was attributed to site masking: A limited population of highly accessible, high-affinity sites was retained and occupied rapidly, whereas the total number of available sites was reduced by interfacial coverage and occlusion. Thus, adsorption kinetics and equilibrium capacity were decoupled by the spatial reorganization of binding sites within the multicomponent composite.

**Figure 9:**
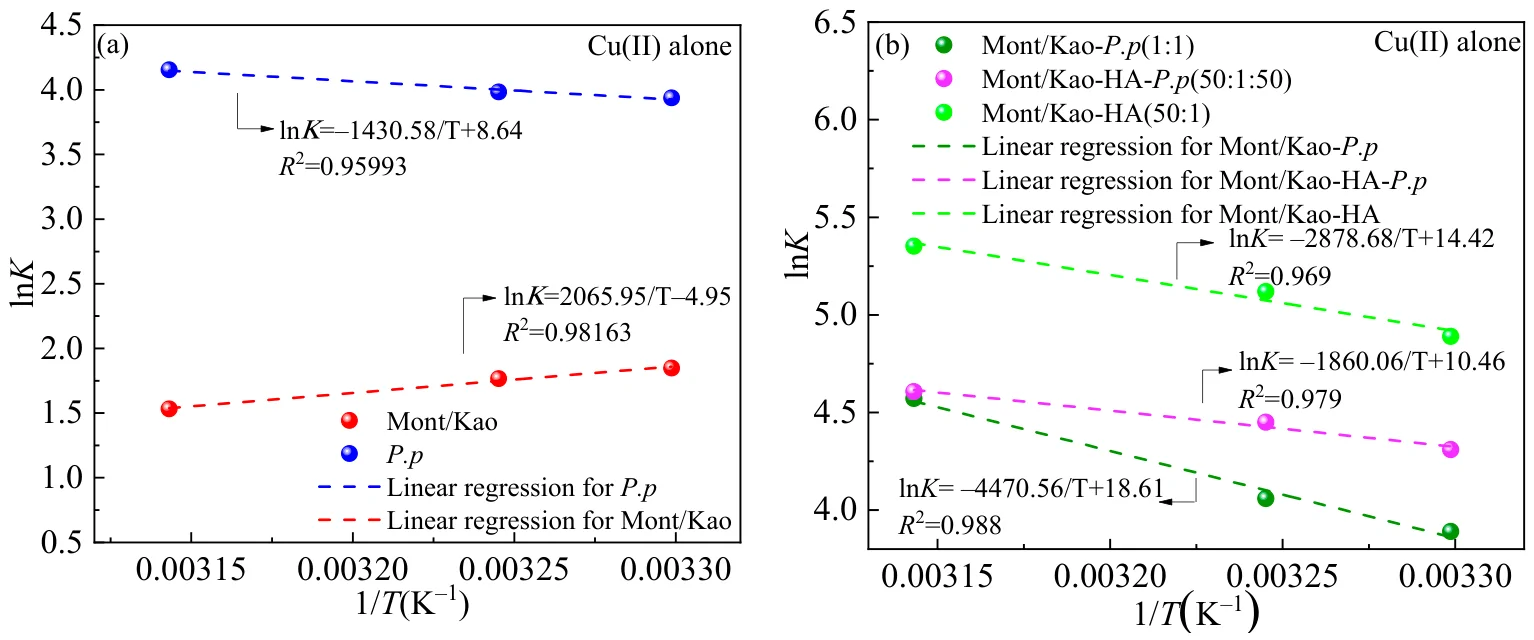
Van’t Hoff plot for Cu(II) adsorption on (**a**) minerals and *P. p*; and (**b**) complexes.

The adsorption isotherms of Cu(II) and Cd(II) on mineral–organic composites under both single-metal and competitive conditions are shown in Figure 8, with the corresponding Langmuir and Freundlich fitting parameters summarized in Table 3. The thermodynamic parameters derived from Van’t Hoff analysis (Figure 9) are presented in Table 4.

In the single-metal system, the Langmuir and Freundlich isotherm models indicated good fits; both models described the adsorption onto Mont/Kao and *P. p*, with correlation coefficients around 0.90 (Figure 8). However, the Mont/Kao-HA, Mont/Kao-*P. p* and Mont/Kao-HA-*P. p* composites all exhibited a typical L-type isotherm, which was better described by the Langmuir model than by the Freundlich one, indicating the adsorption onto a finite number of effective binding sites and an apparent tendency towards monolayer coverage. Similarly, Tian et al. [7] and Chen et al. [44,45,46] demonstrated that the Langmuir model suitably described the adsorption of Pb, Cu, Cd, and Zn on mineral, HA, and *P. p*. In contrast, Xiao et al. [42] and Yang et al. [47] revealed that the Freundlich model offered a better fit for Cry1Ac-Cd(II)/Zn(II) and As(V) adsorption on kaolinite, which assumes multilayer adsorption on the surface.

The adsorption capacity for Cu(II) was substantially higher than that for Cd(II). This difference was attributed to the combined effects of hydration characteristics and metal-ligand affinity, including the smaller hydrated radius of Cu(II) and its stronger affinity for oxygen-containing functional groups, such as carboxyl and phenolic hydroxyl groups, in HA. A similar preferential retention of Cu(II) was reported for montmorillonite-humic acid composites, where Cu(II) was proposed to replace exchangeable Na^+^ and to form stronger surface complexes because of its greater polarizing power and coordination affinity.

The adsorption of Cu(II) onto the pure mineral (Mont/Kao) was an exothermic process (Δ*H*° < 0), characterized by a negative entropy change (Δ*S*° = −41.15 J⋅mol^−1^⋅K^−1^), which is diagnostic of the formation of outer-sphere complexes via ion exchange.

In this mechanism, the metal ion retains its hydration shell, and the reaction is driven by favorable enthalpy from electrostatic attraction. In stark contrast, the adsorption of Cu(II) onto *P. p*, Mont/Kao-HA, Mont/Kao-*P. p*, and Mont/Kao-HA-*P. p* composites was endothermic (Δ*H*° > 0), with large positive entropy changes (Δ*S*° ranging from ~72 to 155 J·mol^−1^·K^−1^). This thermodynamic signature is the hallmark of inner-sphere complexation, where the metal ion must shed its hydration water molecules to form direct coordinate bonds with surface ligands (carboxyl, phosphoryl, hydroxyl groups). The resulting increase in the disorder of the system (Δ*S*° > 0) is the primary driving force for the reaction, overcoming the unfavorable enthalpic cost of dehydration. This finding is in excellent agreement with the pseudo-second-order kinetic model, both pointing unequivocally to chemisorption as the dominant mechanism for all composites containing organic components. Furthermore, the Δ*G*° values for all adsorbents except Mont/Kao were negative, confirming that the adsorption of Cu(II) onto these composites is a spontaneous process (Δ*G*° < 0). The positive Δ*G*° for Mont/Kao indicates the non-spontaneous nature of Cu(II) adsorption on the bare mineral under these specific experimental conditions, making it the least effective sorbent. This finding is entirely consistent with its low equilibrium capacity.

These integrated isotherm, kinetic, and thermodynamic findings demonstrate that the superior performance of mineral–bacterial and ternary composites is not merely an additive effect but arises from a fundamental shift in the adsorption mechanism, from non-spontaneous, outer-sphere complexation on the mineral to spontaneous, inner-sphere chemisorption on the composite. The incorporation of bacteria and humic acid transforms the binding environment, providing a dense array of high-energy coordination sites that enable stronger, more stable metal sequestration.

#### 3.3.2. Adsorption Equilibrium and Thermodynamic Analysis of Competitive Adsorption

**Figure 10:**
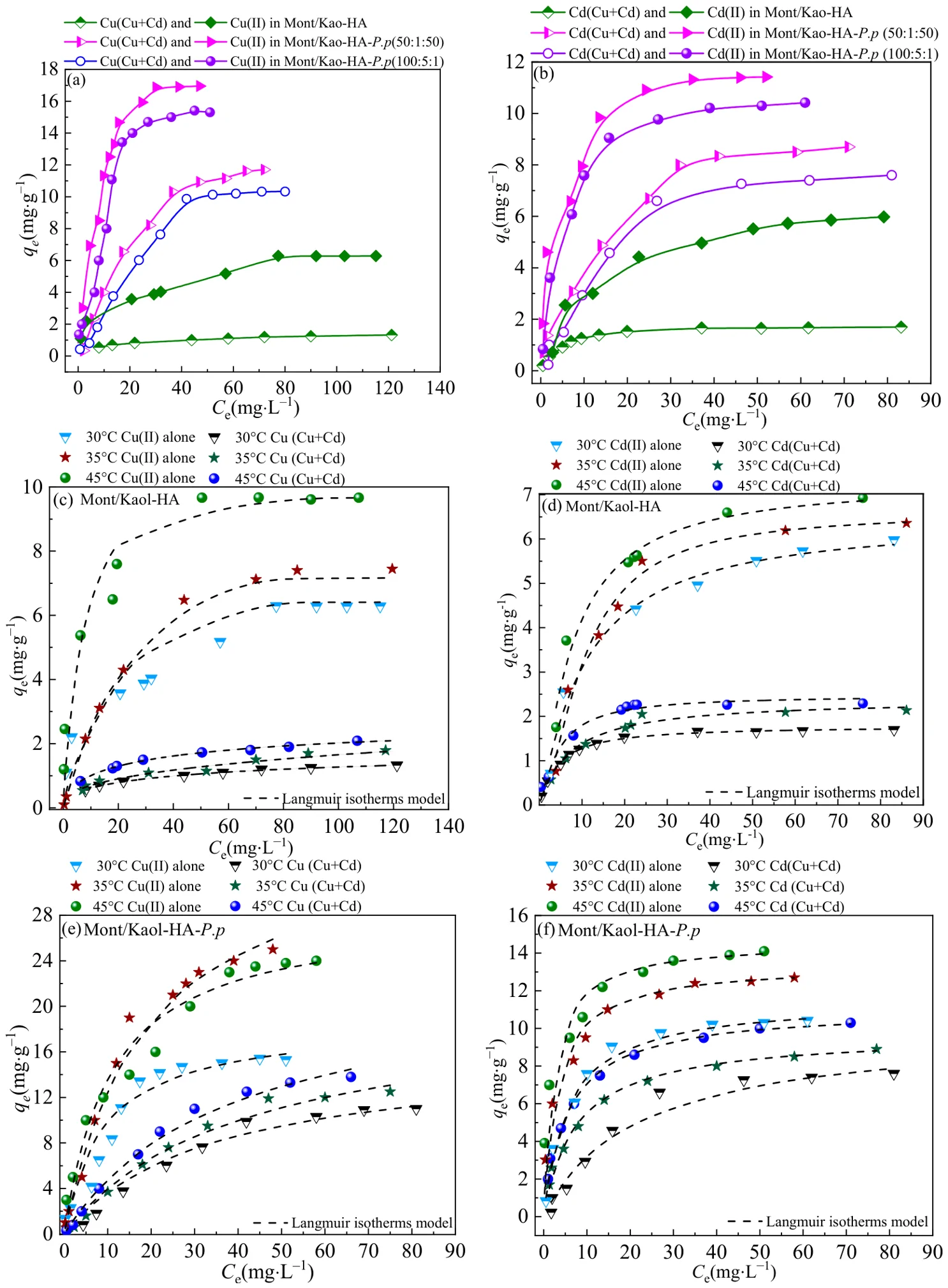
Adsorption isotherms of Cu(II) and Cd(II) on composites at different temperatures. (**a**,**b**) Adsorption isotherms of Cu(II) and Cd(II) on Mont/Kao-HA-*P. p* and Mont/Kao-HA composites; (**c**–**f**) Temperature-dependent adsorption isotherms and model fittings for Cu(II) and Cd(II) adsorption on Mont/Kao-HA and Mont/Kao-HA-P composites.

**Figure 11:**
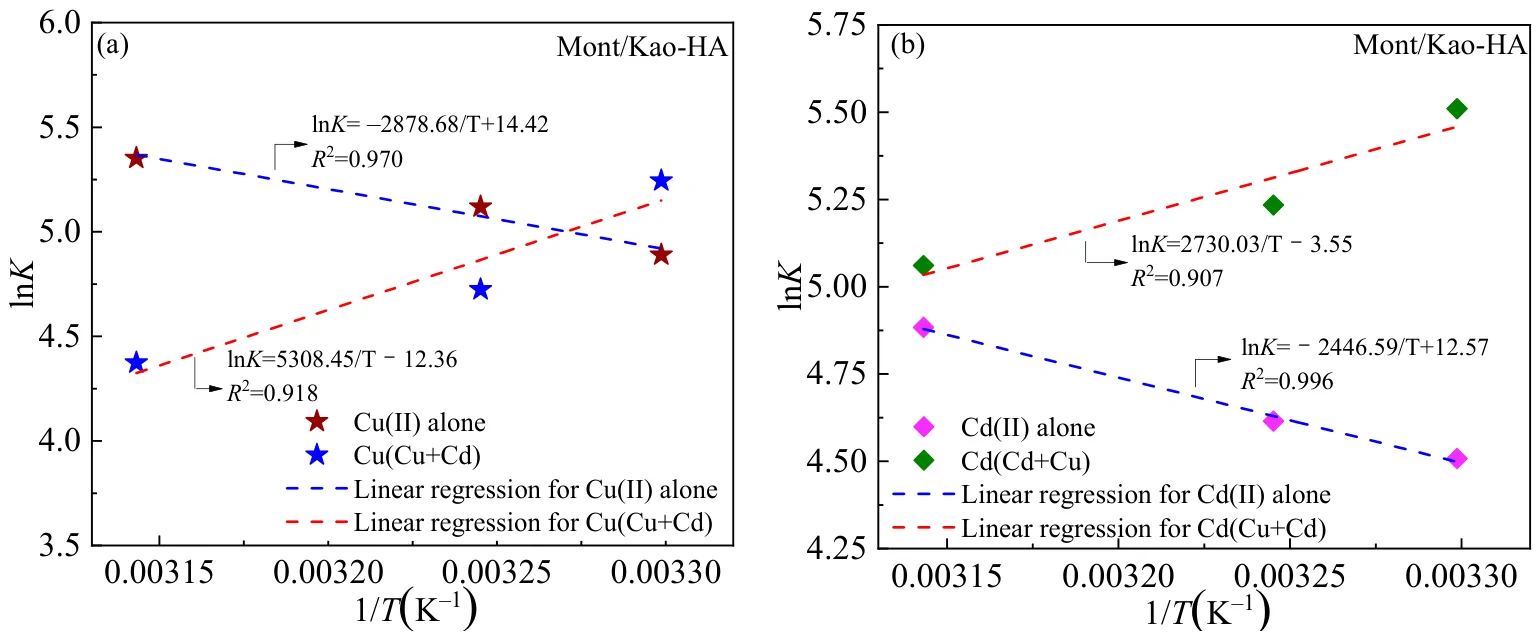

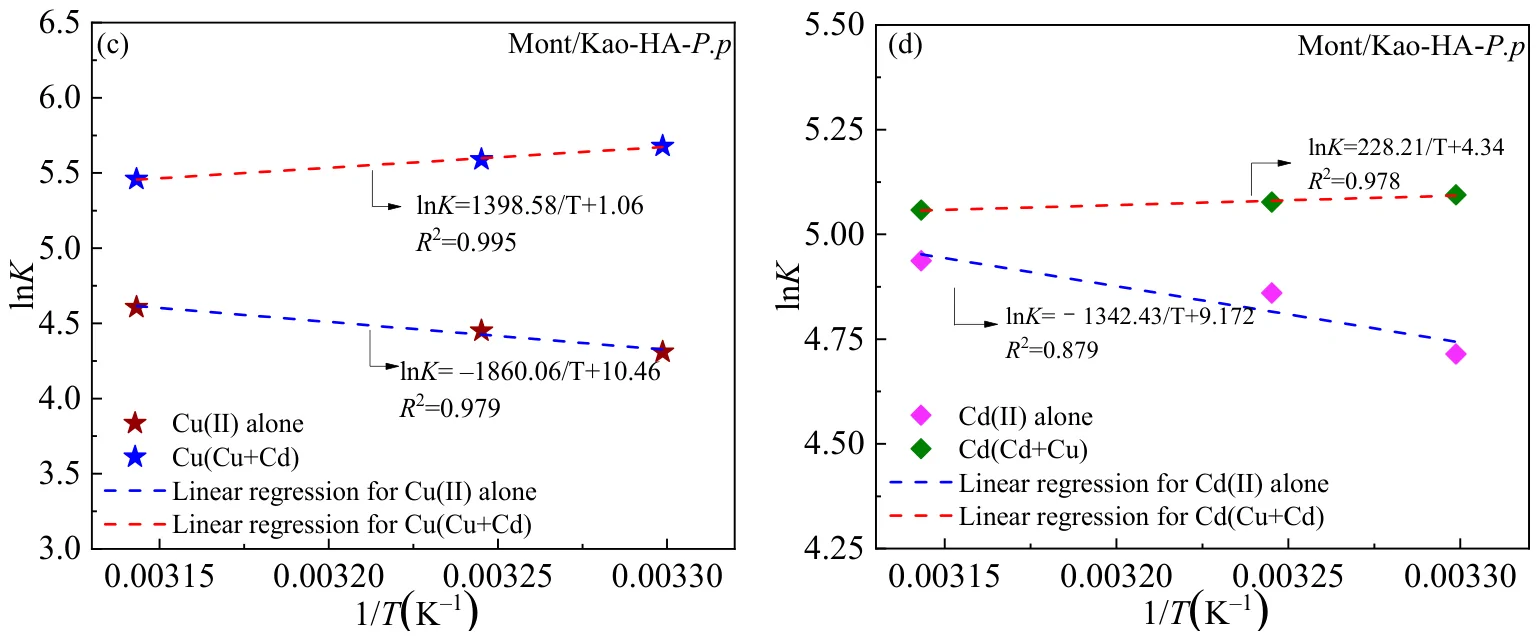
Van’t Hoff plots for the complex adsorption of Cu(II) and Cd(II) on (**a**,**b**) Mont/Kao-HA and (**c**,**d**) Mont/Kao-HA-P composites.

Distinct Cu(II) and Cd(II) binding mechanisms under competitive adsorption conditions are revealed by Figure 10 and Figure 11, together with the isotherm-fitting parameters and the thermodynamic data in Table 4.

Competitive experiments reveal that Cu(II) and Cd(II) compete for a considerable fraction of the available binding sites. For the Mont/Kao-HA composite, the maximum adsorption capacities of both metals were substantially reduced under binary coexistence (Figure 10). Over the temperature range of 30–45 °C, Cu(II) adsorption decreased by 75.5–79.9%, from 6.41–9.66 mg·g^−1^ to 1.32–2.09 mg·g^−1^, whereas Cd(II) adsorption declined by 66.35–71.3%, from 5.99–6.93 mg·g^−1^ to 1.72–2.29 mg·g^−1^. This asymmetric inhibition suggests that competitive sensitivity is governed not only by single-solute adsorption capacity, but also by the extent of overlap between metal-binding sites and the relative affinities of Cu(II) and Cd(II) for different mineral- and HA-associated adsorption domains. Although Cu(II) preferentially occupies high-affinity HA and mineral-edge sites, Cd(II) can compete for these sites while retaining access to permanent-charge and interlayer exchange domains in montmorillonite. Consequently, Cd(II) may follow dual adsorption pathways involving both surface complexation and ion exchange, whereas Cu(II) depends more strongly on a limited population of specific organic and mineral-edge sites. Occupation or shielding of these sites therefore causes a disproportionately large decrease in Cu(II) adsorption.

Competitive adsorption was also influenced by changes in surface charge and composite structure. Initial binding of either metal neutralizes the negative surface charge and weakens the electrostatic driving force for subsequent adsorption. In addition, divalent-metal bridging may induce HA contraction or aggregation, thereby reducing the accessibility of internal carboxyl groups. The observed competition therefore results from a combination of direct site occupation, electrostatic screening, interlayer exchange, and metal-induced reorganization of the mineral-HA interface.

The Langmuir model generally provided a satisfactory description of adsorption by the Mont/Kao-HA composite, indicating a finite effective adsorption capacity. However, the good fit should not be interpreted as evidence of a chemically uniform surface, because the composite contains heterogeneous mineral exchange sites, edge hydroxyls, and HA functional groups. The simultaneous decrease in maximum adsorption capacity and increase in the apparent affinity constant in some competitive systems can be interpreted as an affinity-capacity trade-off. Competition removes many weak and intermediate-energy sites from the adsorption response, leaving a smaller population of highly selective sites. Therefore, the remaining ions may exhibit a high apparent affinity despite the pronounced loss of total adsorption capacity.

The thermodynamic results further reveal that coexistence altered the energetic balance of adsorption. For the Mont/Kao-HA composite, single-metal adsorption was endothermic, with positive Δ*H*° and Δ*S*° values. This behavior is consistent with partial dehydration of Cu(II) and Cd(II) and the release of hydration water and counterions during specific surface complexation. Under competitive conditions, Δ*H*° became negative, and Δ*S*° also decreased markedly. These changes suggest that competition reduced the contribution of dehydration-dependent inner-sphere coordination and increased the relative importance of ion exchange, electrostatic association, and hydrated outer-sphere interactions at permanent-charge sites. The negative entropy changes may further reflect a more ordered arrangement of hydrated ions, interfacial water, and HA chains around the residual adsorption domains.

Nevertheless, the signs of Δ*H*° and Δ*S*° alone cannot uniquely identify inner-or outer-sphere complexes, because the measured thermodynamic parameters represent the combined effects of metal dehydration, counterion release, proton exchange, HA conformational changes, and interfacial-water reorganization. The thermodynamic data therefore support a competition-induced shift in the dominant adsorption pathway, rather than a complete conversion from one exclusive binding mode to another. The negative Δ*G*° values in all systems confirm that adsorption remained spontaneous, although spontaneity did not prevent a substantial reduction in capacity under competitive conditions.

The ternary Mont/Kao-HA-*P. p* composite showed a less pronounced competitive decrease than the Mont/Kao-HA binary composite. This suggests that the chemically heterogeneous ternary interface gave rise to partially differentiated adsorption domains. Cu(II) could preferentially bind to bacterial carboxylate and phosphate groups and to HA ligands, whereas Cd(II) could be distributed between organic functional groups and mineral exchange sites. Such site partitioning reduced direct competition and allowed the ternary composite to retain a larger proportion of its single-metal adsorption capacity.

The composition of the ternary composite was also critical. The formulation with higher mineral and HA contents exhibited better adsorption than that with a larger bacterial fraction. Increasing bacterial biomass did not necessarily increase the number of accessible sites because HA coating and cell aggregation could mask bacterial functional groups and occlude mineral edges. In contrast, a mineral-rich framework preserved exchange domains and structurally separated organic components, while HA supplied additional selective ligands. Therefore, the superior performance of the mineral- and HA-rich composite resulted from a more favorable balance between site density and site accessibility.

Although competition reduced the adsorption of both metals, the ternary composite retained a clear preference for Cu(II) over Cd(II), confirming the stronger coordination of Cu(II) with oxygen- and phosphorus-containing ligands. Importantly, the preferential adsorption of Cu(II) and its stronger relative inhibition in the Mont/Kao-HA system are not contradictory. Selectivity describes the final adsorption of one metal relative to another, whereas inhibition represents the proportional loss relative to its corresponding single-metal system. Cu(II) may therefore remain preferentially retained while suffering a greater relative decrease because its adsorption depends more strongly on a limited number of high-affinity sites.

Overall, these results demonstrate that competitive adsorption at mineral–organic–microbial interfaces is governed by both metal-specific chemistry and composite architecture. Composite formation does not simply add the capacities of individual components; instead, it redistributes, exposes, or masks adsorption sites and thereby changes metal selectivity and binding pathways. More importantly, competitive ions alter not only the adsorption capacity but also the relative contributions of specific coordination, ion exchange, electrostatic association, hydration, and interfacial restructuring.

### 3.4. Characterization of Mineral–Organic Aggregates

As demonstrated above, the affinity and capacity of the composites for heavy metals were primarily regulated by the structural organization of mineral–organic interfaces. To elucidate the mechanisms underlying metal retention, the clay minerals and their corresponding mineral–organic composites were characterized before and after adsorption using XRD, FTIR, SEM and optical microscopy.

#### 3.4.1. Spectral Evidence for Intercalation and Surface Complexation

**Figure 12:**
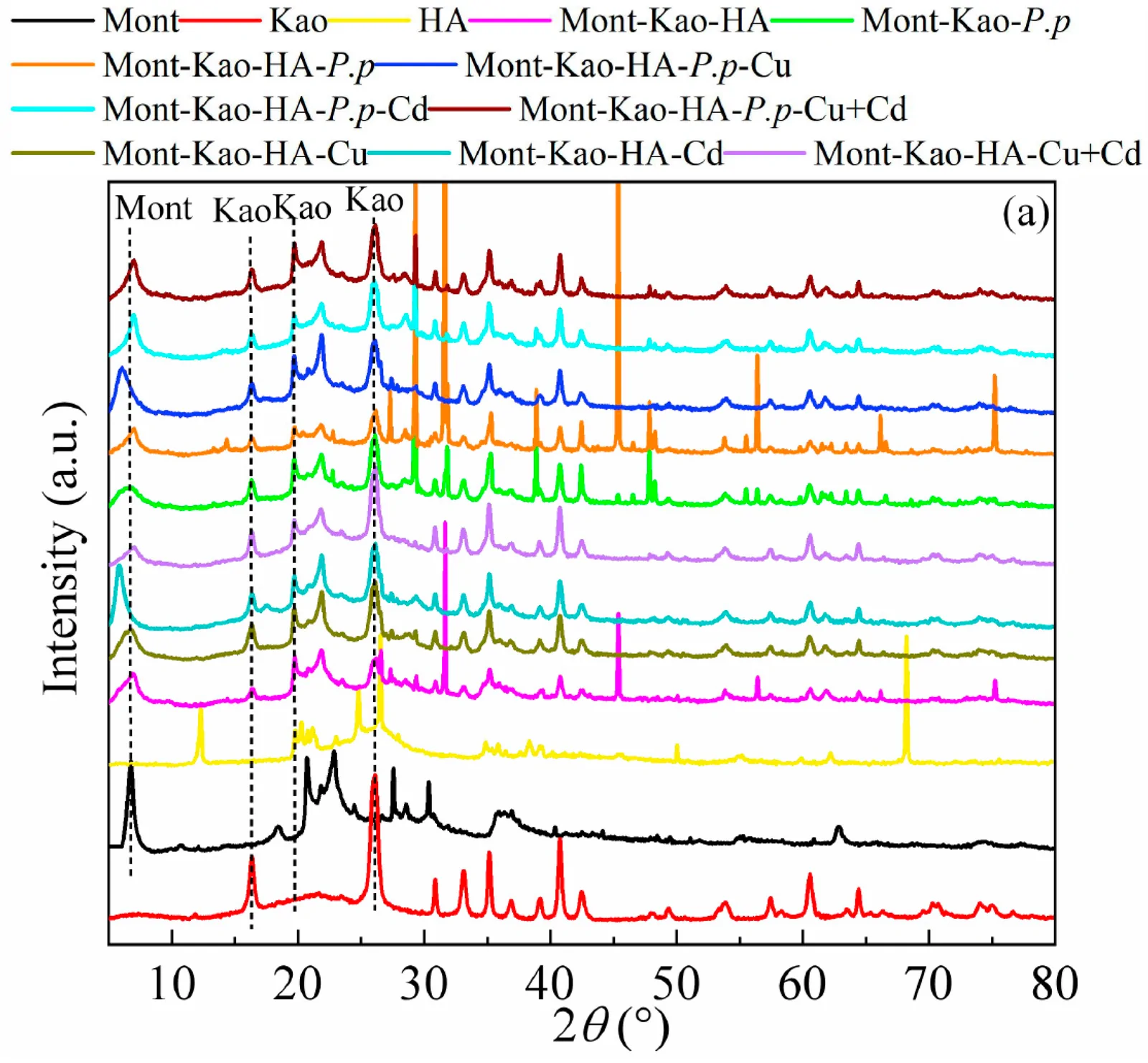

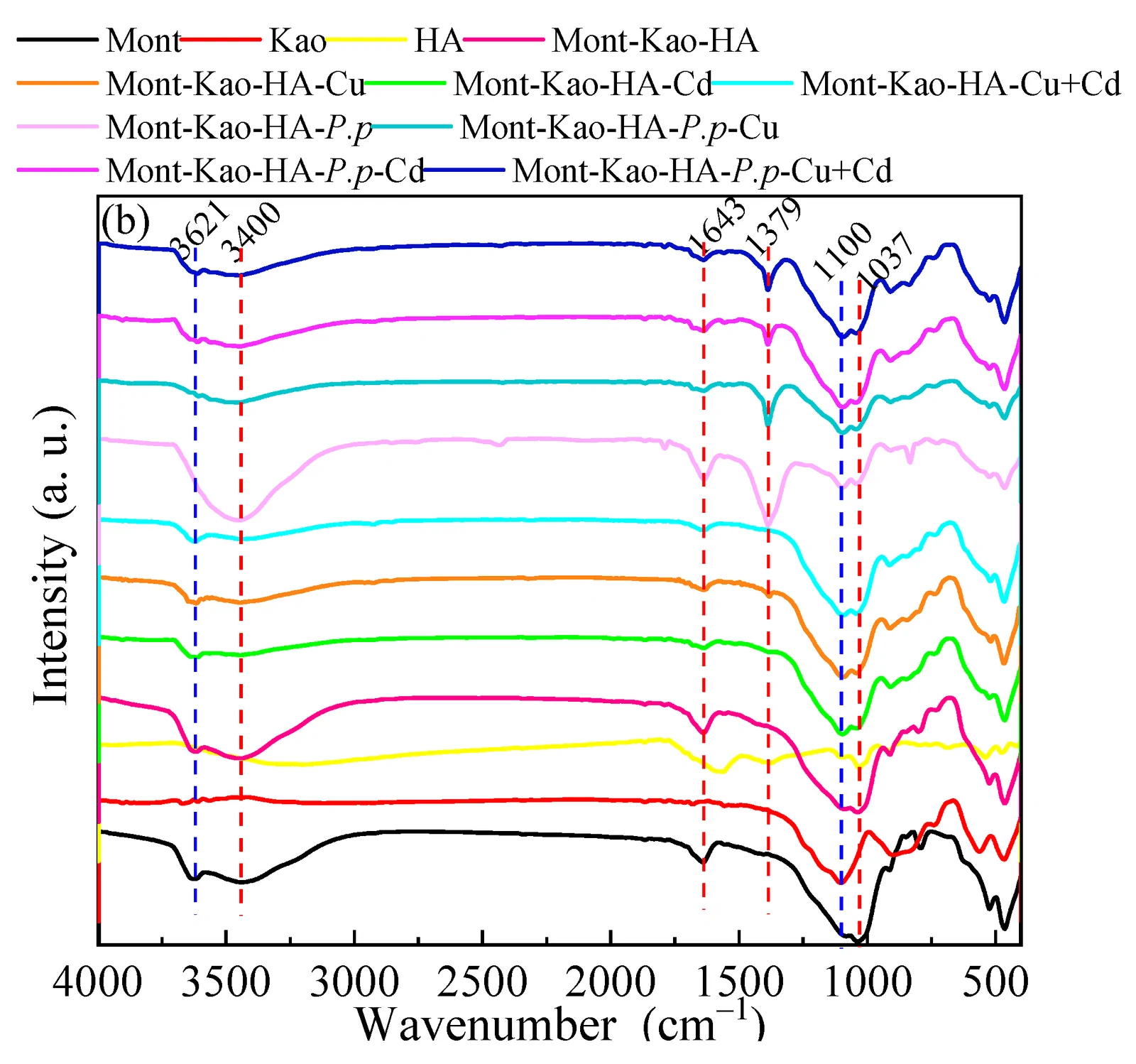
XRD patterns (**a**) and FTIR spectra (**b**) of Mont/Kao-HA composites before and after reaction with heavy metals.

The XRD patterns in Figure 12a demonstrated that the association of HA and bacterial components with the mineral phases was regulated by the contrasting structural characteristics of Mont and Kao. A *d*_001_ spacing of 1.27 nm was observed for pristine Mont, consistent with hydrated Na-dominated montmorillonite, whereas the Kao basal reflection occurred at approximately 0.77 nm, corresponding to its non-expandable 1:1 layer structure. No detectable shift in the characteristic reflections was observed after HA incorporation into the Mont/Kao composite. Intercalation of intact HA molecules into the clay interlayers was therefore not supported, and HA was instead considered to have been associated predominantly with external basal surfaces, edge sites and interparticle domains through hydrogen bonding, ligand exchange, electrostatic interactions and cation bridging [36,48]. Although interlayer expansion was not induced, the surface charge distribution, aggregation behavior and accessibility of reactive sites may still have been modified by the HA coating, which was also reported by Chassapis et al. [49].

Following bacterial incorporation, more pronounced structural changes were observed. The d_001_ spacing of Mont was increased to 1.36 nm in the Mont/Kao-*P. p* composite. The d_001_ value of Mont increased from 1.25 nm (pristine) to 1.36 nm (composite with *P. p*). This expansion is consistent with the intercalation of a monolayer of water and exchangeable cations, possibly influenced by organic fragments derived from bacterial EPS or cell lysis [28,50,51,52]. Because penetration of intact bacterial cells into montmorillonite interlayers is physically unlikely, this expansion was more reasonably attributed to the incorporation of bacterial-derived organic components, including cell-wall fragments, extracellular polymeric substances (EPS) and soluble metabolites, or to changes in interlayer hydration and exchangeable-cation configuration. Thus, bacterial incorporation was considered to have promoted reorganization of the hydrated mineral interface rather than simple attachment onto external surfaces. Changes in the Kao diffraction features following *P. p* addition further suggested species-dependent effects on mineral aggregation and interfacial hydration [40,51], which may have been associated with differences in the distribution of carboxyl, phosphoryl and hydroxyl functional groups within bacterial envelopes and EPS.

The ternary composites exhibited a distinct structural response. No resolvable displacement of the characteristic mineral reflections was detected in the Mont/Kao-HA-*P. p* system, indicating that the pre-established HA layer strongly regulated subsequent mineral–bacteria interactions. Through HA-mediated coating, mineral-microbe contact, local charge distribution and steric accessibility were modified. Consequently, bacterial functional groups may have been incorporated into the composite matrix, whereas a proportion of high-affinity sites may have been masked by the HA coating. This structural constraint provides a mechanistic explanation for the non-additive adsorption behavior observed in the ternary system and agrees with the site-masking effect reported for mineral-HA-microbial composites [40,42,51].

Further structural evidence was obtained after metal adsorption. In the Mont/Kao-HA composite, the Mont d_001_ spacing increased from 1.27 nm to 1.33 nm and 1.53 nm after Cu(II) and Cd(II) adsorption, respectively. This indicates the incorporation of hydrated metal species into the expandable interlayer, with exchange against interlayer Na^+^, Ca^2+^ or other cations likely contributing to retention. The larger expansion induced by Cd(II) was considered to result primarily from differences in hydration behavior, coordination environment and interlayer organization rather than ionic radius alone. In contrast, no significant expansion of Kao was observed after Cu(II) or Cd(II) adsorption, suggesting that metal retention by Kao occurred mainly through interactions with external surfaces and amphoteric edge sites [53,54,55]. These contrasting responses highlight that adsorption performance was determined not only by mineral abundance, but also by the accessibility and spatial distribution of reactive domains.

No measurable change in basal spacing was observed for the Mont/Kao-HA-*P. p* composite following competitive Cu(II)-Cd(II) adsorption. Under these conditions, extensive interlayer rearrangement appeared to contribute less to metal retention, while external organic–mineral and microbial interfaces likely became increasingly important. Competition for exchange sites may have restricted interlayer incorporation, whereas coordination with HA- and EPS-derived functional groups may have contributed more substantially to metal binding. However, unchanged d_001_ values should not be interpreted as direct evidence for a specific transition from inner-sphere to outer-sphere complexation, because both mechanisms may occur without measurable basal expansion. Instead, the XRD results indicate a reduced contribution of large-scale interlayer reorganization under competitive conditions. When considered together with thermodynamic data, a redistribution of metal retention from interlayer-dominated processes towards heterogeneous external binding domains was supported.

The FTIR spectra (Figure 12b) provided complementary molecular-level evidence for these structural changes. The persistence of Si-O stretching bands at approximately 1100 cm^−1^ for Kao and 1037 cm^−1^ for Mont indicated that the aluminosilicate frameworks remained structurally stable during composite formation and metal adsorption. In contrast, changes were observed in bands associated with the organic components. The increased intensity near 1379 cm^−1^ and the shift in the band from approximately 1636 to 1643 cm^−1^ suggested modifications in the chemical environment of aliphatic and carbonyl/carboxylate-containing groups. The involvement of oxygen-containing HA functional groups in mineral association and metal coordination was therefore supported.

Because the 1636–1643 cm^−1^ region may contain contributions from carboxylate stretching, conjugated carbonyl groups, and adsorbed water, the observed spectral shift cannot be assigned exclusively to a single binding mechanism. The FTIR spectrum showed a shift in the asymmetric stretching vibration of carboxylate (νas(COO^−^)) from 1635 cm^−1^ to 1620 cm^−1^ after Cu(II) adsorption, indicating coordination with the carboxyl group. The calculated Δν (νas(COO^−^)—νs(COO^−^)) value decreased from 180 cm^−1^ to 165 cm^−1^, suggesting a shift towards a bidentate or bridging coordination mode for Cu(II) [19,55,56]. For Cd(II), a smaller shift was observed, implying a weaker or predominantly monodentate interaction. Nevertheless, when integrated with the XRD results and adsorption behavior, the participation of deprotonated carboxyl groups, hydrogen bonding at hydroxylated mineral edges and cation-mediated bridging among mineral, HA and bacterial surfaces was indicated. Competitive Cu(II)-Cd(II) adsorption was therefore governed not simply by the loss of available sites, but by redistribution among binding domains differing in accessibility, affinity, and coordination environment. By integrating mineral exchange sites, humic functional groups and microbial interfaces within a single aggregate structure, the composite system provided a more representative model of heterogeneous soil reactive interfaces than conventional single-component sorbents.

#### 3.4.2. Morphological Insights from SEM and Optical Microscopy

**Figure 13:**
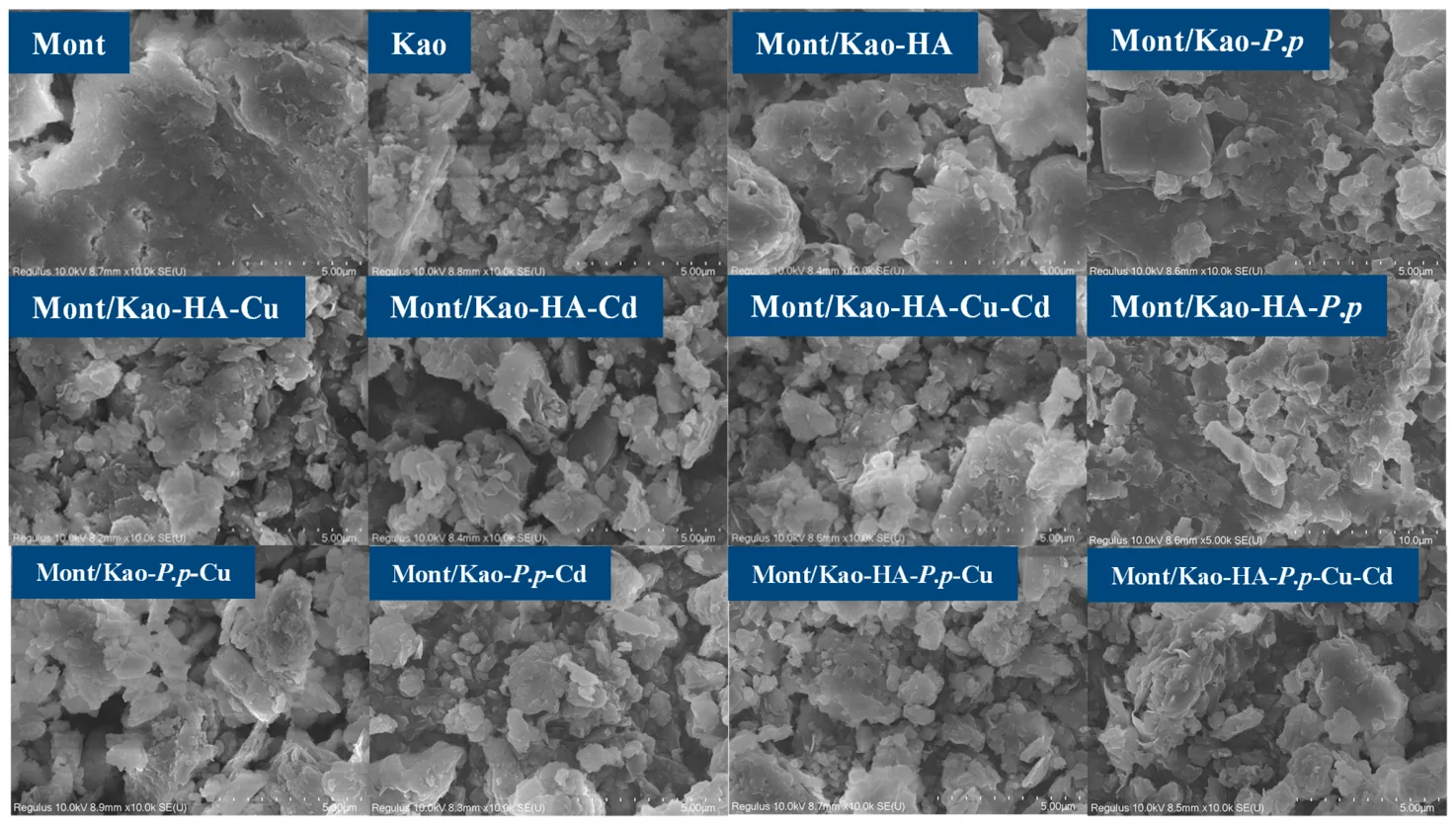
SEM of Mont/Kao–HA–microbe composites before and after heavy metal adsorption.

**Figure 14:**
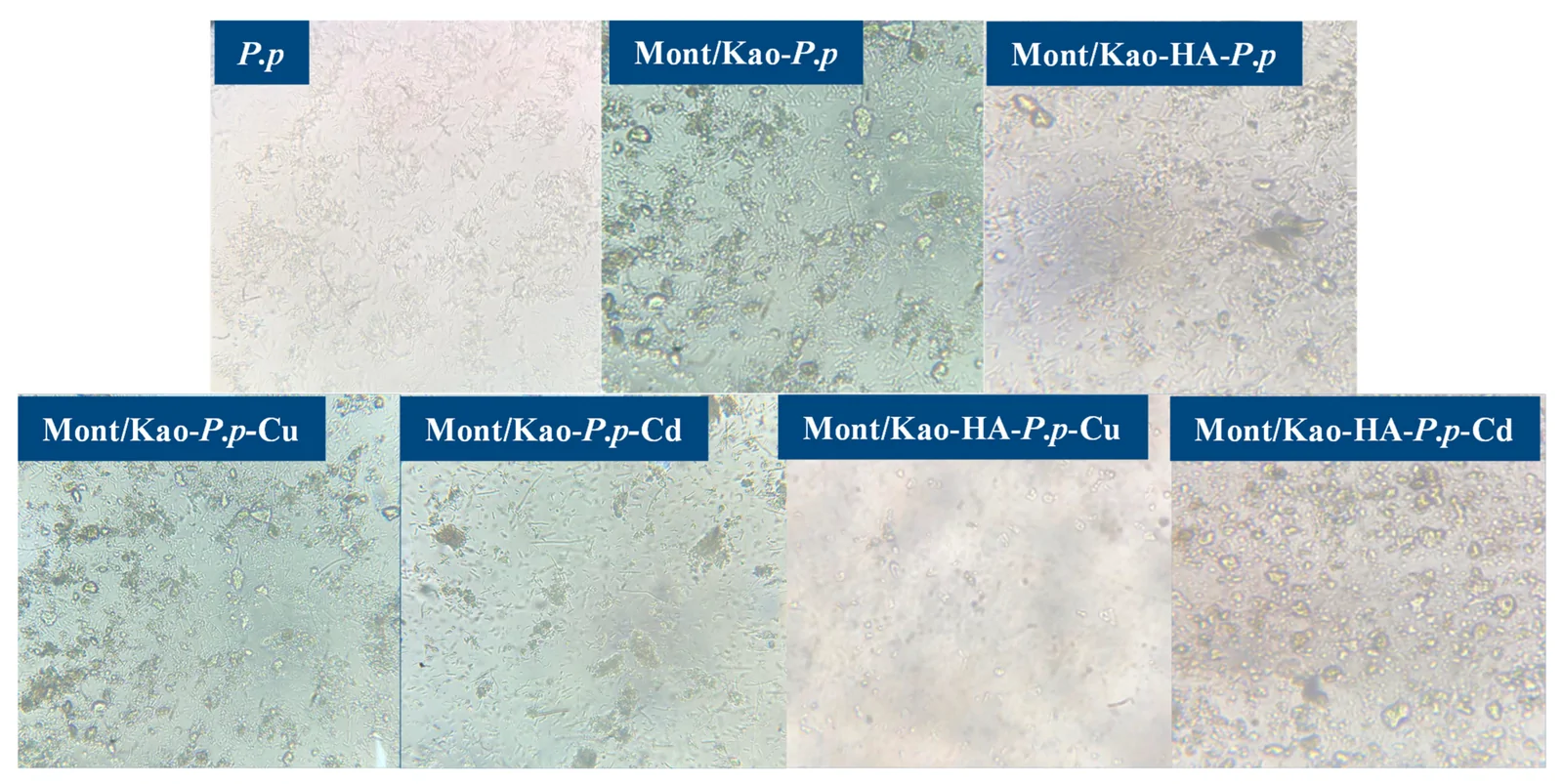
Optical microscopy images of composites before and after heavy metal adsorption.

The SEM images (Figure 13) and optical microscopy observations (Figure 14) provided morphological evidence supporting the structural interpretations derived from XRD and FTIR analyses. Prior to organic modification, the Mont/Kao matrix was characterized by irregularly stacked clay lamellae, relatively smooth surfaces and abundant interparticle voids, resulting in a loosely aggregated structure with accessible external and edge reactive sites. Following HA incorporation, the boundaries of individual clay platelets became less distinct and an amorphous coating was formed, indicating surface coverage, particle bridging and partial pore filling. Although the apparent macroporosity was reduced, additional reactive domains were introduced through HA-derived oxygen-containing functional groups [10,31,42]. Thus, HA modification resulted in a dual effect, whereby surface chemical functionality was enhanced while the accessibility of some pre-existing mineral and microbial binding sites was reduced.

Following the incorporation of *P. p*, bacterial cells preferentially colonized mineral surfaces and interparticle voids, accompanied by EPS accumulation. These processes increased surface heterogeneity and promoted the development of an interconnected microbe-mineral network. The resulting intimate contact between microbial biomass and mineral phases brought carboxyl, phosphoryl, and hydroxyl functionalities into close proximity to mineral reactive sites, thereby enhancing the density of potential interfacial interaction sites. Consequently, enhanced metal retention may have been promoted through both increased ligand availability and local enrichment of metal ions within confined interfacial regions. The microbial component was therefore considered not only as an additional sorption phase, but also as a structural regulator controlling metal distribution within the aggregate.

After Cu(II) and Cd(II) adsorption, increased surface roughness and structural compaction were observed in all composites. Granular and flocculent features were detected on mineral surfaces and bacterial residues, suggesting enhanced aggregation, cation bridging and the development of metal-enriched organic domains. However, the formation of discrete metal hydroxide or carbonate precipitates could not be confirmed solely from SEM morphology. Because secondary-electron SEM provides mainly topographical information, these features may also represent metal–organic complexes, reorganized HA domains, condensed EPS or residual mineral salts. Further confirmation would require complementary elemental and chemical-state analyses.

Within the Mont/Kao-HA composite, Cu(II) and Cd(II) may interact with oxygen-containing functional groups of HA and reactive sites on the mineral surfaces, thereby strengthening interfacial associations within the composite. Such interactions could involve, but are not limited to, coordination with carboxyl groups and metal-mediated bridging. These processes may contribute to the increased structural compactness observed after adsorption; however, the specific coordination environments of Cu(II) and Cd(II) cannot be determined without direct structural evidence such as EXAFS. Although such reorganization may contribute to short-term metal immobilization, excessive pore constriction may restrict diffusion pathways and reduce accessibility to internal reactive sites. The observed morphological evolution therefore reflects a balance between the generation of additional binding domains and the limitation of mass transfer.

In the Mont/Kao-*P. p* composite, deformation or collapse of some bacterial cells was observed after metal exposure. This feature may have resulted from metal-induced cellular stress, dehydration during sample preparation, or a combination of both, and should therefore be interpreted cautiously. Meanwhile, increased accumulation of amorphous materials around cells and mineral particles was observed, consistent with EPS redistribution or enrichment. Through metal binding and the formation of extracellular diffusion barriers, EPS may provide partial protection against metal stress. However, enhanced microbial association does not necessarily indicate irreversible immobilization, because the stability and mobility of metal-EPS complexes may vary under changing soil conditions, including pH, ionic strength and dissolved organic carbon concentrations.

The most integrated architecture was observed in the Mont/Kao-HA-*P. p* ternary composite. Clay platelets were retained as the structural framework, while HA formed a non-crystalline, spatially heterogeneous interfacial matrix coating and connecting the mineral surfaces and bacterial cells, consistent with the intrinsically disordered and chemically heterogeneous nature of humic substances. A tortuous pore network was thereby generated, in which multiple sorption domains were spatially coupled. The non-additive adsorption behavior of the ternary composite originates from the synergistic coupling among the three components. The components are arranged in a complementary manner, where one component provides adsorption sites, another enhances interfacial interactions, and the third improves active-site accessibility by regulating the local environment. This multiscale structural integration creates additional adsorption interfaces and promotes cooperative adsorption interactions, resulting in adsorption performance beyond the simple additive contribution of individual components. Although additional binding sites and interfacial domains were introduced through HA and bacterial incorporation, partial masking of high-affinity mineral and microbial sites was simultaneously induced by the HA coating. Furthermore, aggregation and metal-induced compaction may have restricted access to internal sorption domains. The intermediate adsorption capacity of the ternary system was therefore attributed to the balance between competing processes, whereby ligand enrichment, cation bridging and local metal accumulation were counteracted by steric shielding, pore limitation and competition among Cu(II), Cd(II) and native exchangeable cations.

### 3.5. Adsorption Mechanism and Limitations

When the XRD, FTIR, SEM and optical microscopy results were considered collectively, a multiscale model of competitive metal retention was established. The observed metal adsorption behavior can be interpreted within the framework of well-established mineral-, organic-, and microbe-associated mechanisms. At the crystallographic scale, the higher uptake capacity of montmorillonite is consistent with its expandable interlayers and permanently charged exchange sites, whereas metal retention by kaolinite is expected to occur predominantly on external basal surfaces and edge domains. At the molecular scale, HA and bacterial functional groups provide additional coordination environments through carboxylate-, phosphoryl-, and hydroxyl-mediated interactions. At the aggregate scale, the accessibility of these reactive domains can be further modified by surface coating, particle bridging, pore infilling, and microbial encapsulation. Taken together, our results suggest that metal adsorption in the studied composites reflects the combined influence of these established processes, with adsorption behavior depending not only on the abundance of individual reactive components but also on their spatial arrangement and accessibility within the composite structure.

The mineral–organic–microbial associations should be interpreted as the result of interfacial reorganization processes rather than as additive combinations of independent sorbents. HA was proposed to act simultaneously as a source of metal-binding ligands, a bridging phase between mineral particles and a masking layer over pre-existing high-affinity sites. Additional ligands and extracellular polymeric substance-rich domains were introduced through bacterial incorporation; however, their accessibility was controlled by the surrounding mineral-HA architecture. Competitive Cu(II)-Cd(II) adsorption was likely regulated by a hierarchy of structurally distinct sites, among which interlayer ion exchange, external-surface complexation and binding by organic ligands may have contributed to different extents according to composite configuration. Through this framework, the apparently contradictory observation that greater chemical complexity did not necessarily result in a proportional increase in adsorption capacity can be reconciled.

In soils, clay minerals are commonly incorporated into organo-mineral and microbe-associated aggregates rather than occurring as isolated particles. By combining expandable and non-expandable clay minerals, HA, and bacterial cells within a single experimental system, a closer representation of the heterogeneity of natural soil interfaces was achieved than would be provided by a purified single-component sorbent. The distinction between total sorption capacity and effective site accessibility is particularly important for remediation design. Materials with a high nominal density of functional groups may not necessarily exhibit the greatest effective capacity when aggregation, competitive occupation, and steric effects could influence the accessibility of reactive sites. Accordingly, the optimization of mineral–organic composites should be directed not only towards increasing ligand abundance, but also towards maintaining pore connectivity, interlayer accessibility and exposure of high-affinity humic and microbial binding domains.

Several limitations should nevertheless be acknowledged. The characterization methods used here provided predominantly static, ex situ representations of the composites, and dynamic structural changes occurring during adsorption, including progressive rearrangement of HA and bacterial extracellular polymeric substances under increasing metal loading, were not resolved. In addition, the nanoscale distribution of HA and bacterial components on individual clay particles could not be determined by conventional XRD or FTIR. Furthermore, the different chemical forms of retained metals, including specifically adsorbed species and surface precipitates, could not be distinguished from SEM observations alone. Future investigations should therefore incorporate higher-resolution and spatially resolved techniques, including TEM coupled with EDS and SR-μXRF, to determine the spatial relationships among mineral phases, organic components and retained metals at the single-particle scale. Despite these constraints, a coherent conceptual framework was established through which the structure–function relationships of mineral–organic composites can be interpreted and their design for heavy-metal immobilization can be more effectively optimized.

These findings provide a mechanistic basis for the potential application of the investigated system in contaminated agricultural soils. Nevertheless, the heterogeneity of real soils cannot be fully reproduced by the laboratory composite system, as treatment performance may be affected by variations in physicochemical properties, contaminant distribution and speciation, microbial communities, and environmental conditions. Therefore, further validation using heterogeneous soils and under field-relevant or in situ conditions is needed to assess the robustness and practical applicability of the proposed approach.

## 4. Conclusions

This study provides new insights into the competitive adsorption behavior of Cu(II) and Cd(II) within multi-component montmorillonite/kaolinite-humic acid-*Pseudomonas putida* composites, demonstrating that interfacial composition, spatial distribution of reactive sites, and metal-metal interactions collectively regulate heavy metal retention. Among the investigated components, bacterial biomass exhibited a dominant contribution to Cu(II) and Cd(II) immobilization compared with individual mineral or humic acid phases. Under competitive adsorption conditions, bacteria-containing composites, particularly the Mont/Kao-*P. p* (1:1) system, maintained higher metal sorption capacities than the corresponding bacteria-free Mont/Kao-HA systems, with bacterial contributions accounting for more than 50% of total sorption, especially for Cu(II). Humic acid played a concentration-dependent regulatory role in metal adsorption. At relatively low concentrations, HA enhanced mineral dispersion and increased the accessibility of reactive surfaces, thereby promoting metal uptake. However, excessive HA loading reduced adsorption efficiency through surface coverage, steric effects, and pore occupation, which partially restricted the accessibility of high-affinity binding sites, including those associated with bacterial surfaces. Consequently, ternary Mont/Kao-HA-*P. p* composites did not always exhibit superior performance compared with bacteria-mineral binary composites. Competitive interactions between Cu(II) and Cd(II) were strongly asymmetric. The presence of Cd(II) substantially inhibited Cu(II) adsorption, reducing the maximum sorption capacity of Cu(II) by 75.5–77.9% in Mont/Kao-HA systems, and 13.8–14.9% in Mont/Kao-HA-*P. p* systems. In contrast, Cd(II) showed greater adaptability under competitive conditions, likely due to its preferential occupation of additional ion-exchange sites associated with montmorillonite interlayers, which were less favorable for Cu(II) retention. These findings highlight that heavy metal immobilization in complex soil-like matrices cannot be accurately predicted solely from the properties of individual components, as emergent interfacial interactions and competitive effects strongly influence overall sorption behavior.

Overall, this study provides a mechanistic basis for understanding non-additive adsorption behavior in complex soil systems and offers important implications for the remediation and management of heavy-metal-contaminated agricultural soils. From an applied perspective, metal immobilization may be enhanced through the combined use of metal-resistant bacteria and clay minerals, whereas the type and dosage of organic matter should be carefully optimized, as excessive organic matter may mask bacterial adsorption sites and reduce remediation efficiency. In soils co-contaminated with multiple heavy metals, remediation strategies should further account for contaminant-specific adsorption behavior, with particular attention to pH regulation because of its critical role in governing competitive adsorption processes. Future studies should extend these findings from simplified laboratory systems to heterogeneous agricultural soils through the development of mechanistic and multiscale models that explicitly incorporate interactions among minerals, organic matter, and microorganisms. Integrating molecular-scale characterization techniques, such as EXAFS and ITC, with field-based investigations and a broader range of environmentally relevant soil components will further improve the robustness, transferability, and predictive capability of adsorption models. Collectively, these advances will support more reliable predictions of heavy-metal environmental fate and facilitate the development of targeted, efficient, and sustainable soil remediation strategies.

## Figures and Tables

**Table 1:** Composition and preparation conditions of mineral-based composites.

Composite Type	Components	Mass Ratio (Dry Weight Basis)
Mont/Kao	Mont: 2.0 g Kao: 2.0 g	1:1
Mont/Kao-*P. p*	Mineral ratio-based, biomass dry-weight-based	1:1, 10:1, and 100:1 (mineral: *P. p*)
Mont/Kao-HA	Mont/Kao: 2.0 g; HA: 100, 500, 800, 1200, and 1500 mg∙L^−1^; 200 mL	Based on initial-residual HA concentrations
Mont/Kao-HA-*P. p*	Based on mineral: HA: bacteria ratios	50:1:50 and 100:5:1 (mineral: HA: *P. p*)

**Table 2:** Adsorption kinetics parameters for Cu(II) and Cd(II) on mineral, HA and bacteria composites.

Heavy Metal	Model	Parameters	Mont/Kao (1:1)	Mont/Kao-HA (50:1)	Mont/Kao-*P. p*			*P. p*	Mont/Kao-HA-*P. P* (50:1:50)
					1:1	10:1	100:1		
Cu(II)	Pseudo-first-order	*k*_1_ (min^−1^)	0.011	0.010	0.0074	0.011	0.016	0.013	0.0050
		*q*_e_ (mg∙g^−1^)	2.04	1.41	0.61	1.30	3.23	1.24	0.25
		*R* ^2^	0.82	0.69	0.34	0.84	0.98	0.82	0.48
	Pseudo-second-order	*k*_2_ (mg∙g^−1^·min^−1^)	0.012	0.020	0.076	0.043	0.0094	0.047	0.12
		*q*_e_ (mg∙g^−1^)	3.59	4.33	5.78	5.01	4.14	7.03	4.72
		*R* ^2^	0.99	0.99	0.99	0.99	0.99	0.99	0.99
Cd(II)	Pseudo-first-order	*k*_1_ (/min)	0.018	0.0057	0.011	0.0064	0.021	0.74	0.38
		*q*_e_ (mg∙g^−1^)	1.54	1.43	1.23	1.17	1.11	2.09	1.47
		*R* ^2^	0.86	0.65	0.59	0.33	0.73	0.87	0.84
	Pseudo-second-order	*k*_2_ (mg∙g^−1^·min^−1^)	0.033	0.027	0.28	0.26	0.097	0.037	0.043
		*q*_e_ (mg∙g^−1^)	3.19	4.55	5.85	5.22	3.79	6.99	4.75
		*R* ^2^	0.99	0.99	0.99	0.99	0.99	0.99	0.99

**Table 3:** Summary of adsorption isotherm parameters obtained by fitting Langmuir and Freundlich isotherm models.

Absorbent	Metal	System	Langmuir			Freundlich		
			*q*_max_ (mg∙g^−1^)	*b* (L∙mg^−1^)	*R* ^2^	*k*_f_ (mg∙g^−1^)	1/*n*	*R* ^2^
Mont/Kao (1:1)	Cu	Cu alone	7.72	0.10	0.988	1.24	0.43	0.998
	Cd	Cd alone	6.51	0.11	0.998	1.25	0.38	0.879
*P. p*	Cu	Cu alone	32.74	0.18	0.995	2.21	0.47	0.962
	Cd	Cd alone	29.17	0.064	0.975	1.85	0.39	0.886
Mont/Kao-*P. p* (1:1)	Cu	Cu alone	25.48	0.053	0.953	1.63	0.68	0.950
	Cd	Cd alone	18.40	0.065	0.982	1.45	0.60	0.971
Mont/Kao-*P. p* (10:1)	Cu	Cu alone	18.19	0.11	0.960	2.60	0.50	0.970
	Cd	Cd alone	16.04	0.053	0.993	0.99	0.66	0.937
Mont/Kao-*P. p* (100:1)	Cu	Cu alone	12.21	0.055	0.980	0.83	0.62	0.980
	Cd	Cd alone	10.26	0.17	0.997	1.78	0.47	0.892
Mont/Kao-HA (50:1)	Cu	Cu alone	7.08	0.074	0.973	1.27	0.34	0.978
		Cu + Cd	1.49	0.059	0.996	7.74	0.30	0.989
	Cd	Cd alone	7.70	0.11	0.999	1.45	0.52	0.884
		Cu + Cd	2.09	0.152	0.970	2.23	0.23	0.879
Mont/Kao-HA-*P. P* (50:1:50)	Cu	Cu alone	18.50	0.11	0.966	2.31	0.53	0.945
		Cu + Cd	15.75	0.031	0.946	0.46	0.76	0.955
	Cd	Cd alone	11.43	0.19	0.999	1.85	0.49	0.884
		Cu + Cd	9.85	0.049	0.970	0.38	0.77	0.863

**Table 4:** Thermodynamic parameters for the competitive adsorption of Cu(II) and Cd(II) onto mineral, HA and bacteria composites.

Sorbent	Metal	System	Δ*G*° (kJ∙mol^−1^)			Δ*H*° (kJ∙mol^−1^)	Δ*S*° (J∙mol^−1^·K^−1^)
			303.15	308.15	318.15		
Mont/Kao (1:1)	Cu	alone	−4.17	−4.50	−4.09	−17.18	−41.15
*P. p*	Cu	alone	−9.89	−10.24	−10.96	11.89	71.83
Mont/Kao-*P. p* (1:1)	Cu	alone	−9.73	−10.51	−12.05	37.17	154.72
Mont/Kao-HA (50:1)	Cu	alone	−12.41	−13.01	−14.21	23.93	119.89
		Cu + Cd	−13.22	−12.10	−11.57	−44.13	−103.02
	Cd	alone	−11.36	−11.82	−12.92	20.34	104.51
		Cu + Cd	−13.89	−13.41	−13.39	−22.70	−296.35
Mont/Kao-HA-*P. P* (50:1:50)	Cu	alone	−10.90	−11.34	−12.21	15.47	86.96
		Cu + Cd	−14.31	−14.32	−14.44	−11.63	8.81
	Cd	alone	−11.88	−12.45	−13.06	11.16	76.26
		Cu + Cd	−12.84	−13.01	−13.38	−1.90	36.08

## Data Availability

The raw data supporting the conclusions of this article will be made available by the authors upon request.

## Abbreviations

The following abbreviations are used in this manuscript:

SEM	Scanning electron microscope
XRD	X-ray diffraction
FTIR	Fourier transform infrared spectroscopy
Mont	Montmorillonite
Kao	Kaolinite
HA	Humic acid
*P. p*	*Pseudomonas putida*
HM	Heavy metal
AAS	Atomic absorption spectrometry
XAS	X-ray absorption spectroscopy
